# Specific Microbial Taxa and Functional Capacity Contribute to Chicken Abdominal Fat Deposition

**DOI:** 10.3389/fmicb.2021.643025

**Published:** 2021-03-17

**Authors:** Hai Xiang, Jiankang Gan, Daoshu Zeng, Jing Li, Hui Yu, Haiquan Zhao, Ying Yang, Shuwen Tan, Gen Li, Chaowei Luo, Zhuojun Xie, Guiping Zhao, Hua Li

**Affiliations:** ^1^Guangdong Provincial Key Laboratory of Animal Molecular Design and Precise Breeding, Foshan University, Foshan, China; ^2^Guangdong Tinoo’s Foods Group Co., Ltd., Qingyuan, China; ^3^Xianxi Biotechnology Co. Ltd, Foshan, China; ^4^Institute of Animal Sciences, Chinese Academy of Agricultural Sciences, Beijing, China

**Keywords:** chickens, abdominal fat deposition, cecal microbiota, microbial composition, microbial functional, metabolism capacity

## Abstract

Genetically selected chickens with better growth and early maturation show an incidental increase in abdominal fat deposition (AFD). Accumulating evidence reveals a strong association between gut microbiota and adiposity. However, studies focusing on the role of gut microbiota in chicken obesity in conventional breeds are limited. Therefore, 400 random broilers with different levels of AFD were used to investigate the gut microbial taxa related to AFD by 16S rRNA gene sequencing of 76 representative samples, and to identify the specific microbial taxa contributing to fat-related metabolism using shotgun metagenomic analyses of eight high and low AFD chickens. The results demonstrated that the richness and diversity of the gut microbiota decrease as the accumulation of chicken abdominal fat increases. The decrease of Bacteroidetes and the increase of Firmicutes were correlated with the accumulation of chicken AFD. The Bacteroidetes phylum, including the genera *Bacteroides*, *Parabacteroides*, and the species, *B. salanitronis*, *B. fragilis*, and *P. distasonis*, were correlated to alleviate obesity by producing secondary metabolites. Several genera of Firmicutes phylum with circulating lipoprotein lipase activity were linked to the accumulation of chicken body fat. Moreover, the genera, *Olsenella* and *Slackia*, might positively contribute to fat and energy metabolism, whereas the genus, *Methanobrevibacter*, was possible to enhance energy capture, and associated to accumulate chicken AFD. These findings provide insights into the roles of the gut microbiota in complex traits and contribute to the development of effective therapies for the reduction of chicken fat accumulation.

## Introduction

Genetically selected chickens with better growth and early maturation are accompanied by an incidental increase in abdominal fat accumulation (Abdalla et al., 2018). This results in a reduction in the quality of meat that can be considered unhealthy, as well as in an increase in feed cost (Jiang et al., 2017). To date, high-abdominal fat accumulation in commercial broilers hinders profitable farming. In recent years, the focus of research has been on genetic and nutritional regulation of fatty acid synthesis and lipid deposition, and multiple genetic factors, including quantitative trait loci, candidate genes, mRNA, miRNA, and LncRNA, have been identified with the advances in omics technologies (Zhang et al., 2012, 2014; Demeure et al., 2013; Cui et al., 2018; Li H. et al., 2020; Zhang M. et al., 2020).

However, accumulating and emerging lines of evidence from humans (Backhed et al., 2004; Ley et al., 2005), mice (Ridaura et al., 2013), and livestock (Huang et al., 2020) have revealed a strong association between the gut microbiota and adiposity. For instance, the phylum Firmicutes is more abundant in obese than lean individuals, and vice versa, for Bacteroidetes (Ley et al., 2006). In contrast, after a weight loss program for obese individuals, the relative abundance of Bacteroidetes increased and was accompanied by a decrease in Firmicutes (Ley et al., 2006). Furthermore, by transferring gut microbiota from obese or lean mice to germ-free mice, it has been shown that a high Firmicutes to Bacteroidetes ratio increased body fat accumulation (Ley et al., 2005). For chickens, it has been revealed that the long-term divergent selection of chicken with abdominal fat deposition (AFD) not only altered the composition of gut microbiota, but also influenced its functions by enriching its relative abundance in certain microbial taxa (Ding et al., 2016; Hou et al., 2016). Moreover, the gut microbiota has been suggested to be largely independent of host genetics in regulating fat deposition in chickens (Wen et al., 2019). Furthermore, the duodenal and cecal microbiota have a greater contribution to fat deposition and could separately account for 24% and 21% of the variance in the abdominal fat mass after correcting for host genetic effects (Wen et al., 2019). Therefore, the gut microbiota is regarded as an important factor in modulating fat deposition in broiler chickens.

However, most available data are based on human or mammal models, which may not be completely suited in the case of chickens, because of its unique anatomy and physiology. Most currently published studies only describe the structure and function of the chicken gut microbiota (Ding et al., 2016), and the spatial and temporal changes upon specific stimulation resulting from feed additives (Shang et al., 2015), heat stress (Shi et al., 2019; Zaytsoff et al., 2020), and caged/free-range (Chen et al., 2019; Xiang et al., 2021). Meanwhile, the limited studies focusing on the possible contribution of gut microbiota in modulating chicken obesity have mainly examined this aspect using the divergently selected lean and fat broiler chicken lines (Ding et al., 2016; Hou et al., 2016), lacking in the ability to highlight the specific microbiota taxa associated with AFD in conventional chicken breeds.

In this study, the same random flock consisting of 400 broilers differentiated on AFD was used in the Tiannong Partridge Chickens commercial strain. AFD traits and fatty acid composition of all birds were determined in the flock. Based on their abdominal fat percentage (AFP), they were divided into high AFP (HH) and low AFP (LL) groups. Representative samples were then investigated using 16S rRNA gene sequencing to provide a global perspective on the gut microbial taxa related to AFD. Next, samples with extremely divergent AFP traits were subjected to shotgun metagenomic analysis to identify the specific gut microorganisms contributing to fat-related metabolism.

## Materials and Methods

### Chicken and Sample Collection

A random commercial flock of Tiannong Partridge chickens, consisting of 5,000 hens of the same age, was raised free on a farm in Guangdong Tinoo’s Foods Co. Ltd., and fed with a commercial standard diet during the age of 1 to 125 days. Then, on day 126, 400 random hens were collected and slaughtered using the mechanized slaughter line with moderate scalding water temperature. The same part of the pectoral muscle was collected from all hens and the cecum content was randomly collected from 140 chickens. All samples were rapidly frozen using dry ice and stored at −80°C for subsequent analyses.

### Phenotypic Trait Measurements

The phenotypic traits of chickens, including body weight (BW), carcass weight (CW), eviscerated weight (EW), and abdominal fat weight (AFW), were measured on the spot, and the AFP was calculated later. A 2-g sample of each pectoral muscle tissue was homogenized and lipids were extracted following the Folch’s lipids extraction procedure. The contents of intramuscular fat (IMF), triglycerides (TG), phospholipids (PL), and cholesterol (CHO) were measured using commercial kits (Nanjing Jiancheng Bioengineering Institute, Nanjing, China).

### 16S and Shotgun Metagenomic Sequencing

For 76 representative samples, total DNA was extracted from the cecal samples using the QIAamp Fast DNA Stool Mini Kit (QIAGEN, Hilden, Germany). The V3-V4 region of 16S rRNA gene was amplified with primer 341F/806R (341F: CCTACGGGNGGCWGCAG, 806R: GGACTACHVGGGTATCTAAT). The PCR reaction was conducted using Phusion^®^ High-Fidelity PCR Master Mix (NEB, Beverly, MA, United States) with 30 cycles. PCR products were purified using the QIAquick Gel Extraction Kit (QIAGEN). Libraries were generated using the TruSeq^®^ DNA PCR-Free Sample Preparation Kit (Illumina, San Diego, CA, United States) following the manufacturer’s recommendations. Sequencing was conducted on the Illumina HiSeq2500 platform. For a subset of eight individuals, the same DNA extracts were subjected to shotgun metagenomic sequencing. Briefly, qualified genomic DNA was fragmented by sonication to a size of 350bp, and then end-repaired, A-tailed, and adaptor ligated using NEBNext^®^ Ultra^TM^ DNA Library Prep Kit for Illumina (NEB, United States) according to the preparation protocol. DNA fragments with length of 300–400 bp were enriched by PCR. Then libraries were paired-end sequenced on the Illumina HiSeq2500 platform.

### 16S rRNA Gene Data Processing

Paired-end reads were assembled using FLASH v1.2.11 (Magoc and Salzberg, 2011) with a minimum overlap of 10 bp and mismatch error rates of 2%. The QIIME2 pipeline was used for data quality control and analyses (Bolyen et al., 2019). All remaining high-quality reads were aligned and clustered into operational taxonomic units (OTUs) using an open reference OTU picking protocol. Next, chimeras and singletons were filtered from the dataset, and OTUs with an average relative abundance of <10^–6^ were removed from the analysis. The OTU abundance of each sample and taxonomic classification from phylum to species were then determined. Spearman’s correlation was calculated using the psych package (v1.8.4) in R without further multiple testing. Microbes were analyzed at the kingdom, phylum, class, order, family, genus, and species levels.

### Shotgun Metagenomic Data Processing

Shotgun metagenomic sequencing data were quality controlled by requiring a minimum of 4M paired-end reads per sample after Nextera library adaptor removal using Trimmomatic v0.39 (Bolger et al., 2014). Quality control methods were run using default parameters. Then, the clean data were assembled individually using MEGAHIT v1.1.2 (Li et al., 2015) stepping over a k-mer range of 21 to 99 to generate sample-derived assembly. Overall, *de novo* assembly statistics were evaluated as a combination of percent paired or singleton reads realigning to the assembly using BWA v0.7.17 (Li and Durbin, 2009). The unmapped reads of each sample were pooled for re-assembly using MEGAHIT v1.1.2 to generate a mixed assembly. Sample-derived assembly and mixed assembly were combined to obtain the final assembly for further analyses. After quality control, clean reads were used to generate taxonomic profiles with taxonomic classifier MetaOthello v1.0 using reads k-mer signatures of 31bp length (Liu et al., 2018). The open reading frame (ORF) was predicted based on the final assembly contigs (> 500 bp) using MetaGeneMark v3.38 with default parameters (Zhu et al., 2010). The predicted ORFs ≥ 300 bp in length from all samples were pooled and combined based on ≥ 95% identity and 90% read coverage using CD-HIT v4.6 (Li and Godzik, 2006) in order to reduce the number of redundant genes for the downstream assembly step. The reads were re-aligned to the predicted gene using BWA to count read numbers.

### Abundance Calculation and Function Annotation

For 16S sequencing, the qualified OTU data were used to calculate α-diversity metrics using one-way analysis of variance (ANOVA) with the Duncan *post hoc* test using the vegan package v2.5.3. Bray-Curtis dissimilarity was employed as β-diversity measure and principal coordinate analysis (PCoA) plot was generated with the ape package. The different sites were statistically compared using analysis of similarity (ANOSIM) with 10,000 permutations. The *p*-values were adjusted by the false discovery rate (FDR) using the Benjamini-Hochberg method with the p.adjust function in R. To construct the sample classifier in each group, the random forest model was applied using the randomforest package v4.6.12 and pROC package v2.0.1 in R project.

The final gene catalog of shotgun metagenome analyses was obtained from non-redundant genes with a gene read count > 2. Clean reads were used to generate taxonomic profile using Kaiju v1.6.3 (Menzel et al., 2016). Bray-Curtis distance matrix based on gene, taxon and function abundance was calculated to evaluate the microbial community differences between samples (groups). The Welch’s *t*-test, Wilcoxon rank test, Adonis (also called PERMANOVA), and the Anosim test were conducted using R project Vegan package. Differential analyses of genes and taxa were performed using metastats and LEfSe software v1.0 (Segata et al., 2011) based on the mean value of all annotated genes. To predict gene function, all unique ORFs were annotated using DIAMOND v0.9.24 (Buchfink et al., 2015) based on the KEGG (release 94.0), CAZy, and eggNOG 5.0 databases. And Welch’s *t*-test and ANOVA were used to investigate the differences in gut functions including KEGG pathways, CAZy, and eggNOG activities.

To reveal the consistency of the results of the shotgun metagenomic and 16S rRNA gene sequencing, LEfSe software was also used to construct a microbiota classification phylogenetic tree based on the species with an average species abundance greater than 1%.

### Co-occurrence Network Construction

The Co-occurrence network of all annotated genes, depicting the differentially enriched metagenomic microbial taxa and functional capacities with all phenotypes, was constructed according to their Pearson’s correlation coefficient in all samples. Edges with Pearson’s correlation coefficient > 0.8 or < -0.8 and *P* < 0.05 were used to construct the network. The resulting network was visualized with gephi-0.9.2 software.

### Statistical Analyses

The mean ± standard deviation (SD) was calculated for all data. The data on host carcass phenotypes and fatty acid composition were examined for normality and homogeneity of variance. Normally distributed data were analyzed using ANOVA. Duncan *post hoc* test was used to analyze differences among groups when significance (*P* < 0.05) was detected using SPSS 23 (IBM, Armonk, NY, United States). For data that were not normally distributed, Kruskal-Wallis H and *post hoc* tests and Mann-Whitney tests were conducted in SPSS 23. All values with *P* < 0.05 were considered statistically significant.

## Results

### Characterization of Host Phenotypes

**FIGURE 1:**
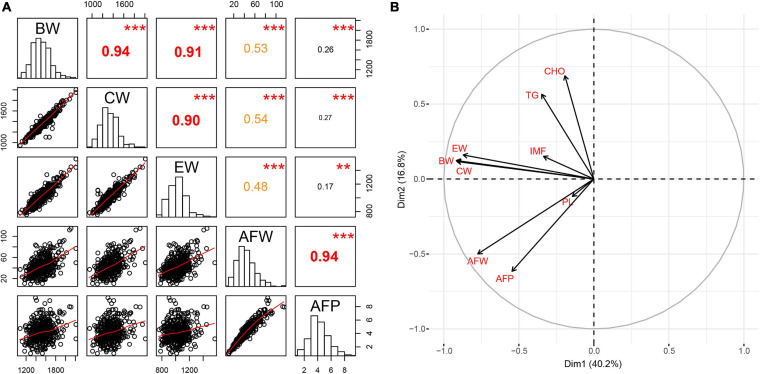
Characterization of host phenotypes. **(A)** The statistic distribution of and correlations among each phenotype, while **(B)** shows the principal components of the carcass traits, the abdominal fat deposition, and the pectoralis lipid composition. BW, body weight; CW, carcass weight; EW, eviscerated weight; AFW, abdominal fat weight; AFP, abdominal fat percentage; IMF, intramuscular fat; TG, triglyceride; PL, phospholipid; CHO, cholesterol. The value in the upper triangular matrix represents the correlation coefficient; two * represents *P* < 0.01, and three * represents *P* < 0.001.

All phenotypic characteristics, including carcass traits (BW, CW, and EW), AFW and AFP and pectoralis lipid composition (IMF, TG, CHO, and PL), fit the normal distribution in this study (Figure 1A and Supplementary Figure 1). Considerable variations were observed regarding both AFW and AFP in the Tiannong Partridge Chickens (Supplementary Table 1). Specifically, the average AFW of Tiannong Partridge Chickens was 43.59 g, and the top 10% of chickens had an average AFW about quadruple over the LL group (77.52 g vs. 19.15 g). Meanwhile, the average AFP was 4.23%, and the top 10% of chickens had an average AFP 3.5 times over the LL group (7.03% vs. 1.98%). The two AFD-related traits, namely, AFW and AFP, exhibited a high correlation (*r* = 0.94, *P* = 0.000) (Figure 1A). AFW was positively correlated with BW, CW, and EW (*r* = 0.47−0.53, *P* < 0.01), and the correlations between AFP and these traits were much weaker but also significant (*r* = 0.16-0.26, *P* < 0.05) (Figure 1A). The pectoralis lipid composition had a weak association with AFD (Supplementary Figure 1). Furthermore, the variable principal component analyses on these phenotypes further suggested that AFD-related traits (AFW and AFP) were relatively independent of carcass traits and pectoralis lipid composition (Figure 1B).

### Correlation Between Gut Microbial Composition and Abdominal Fat Deposition

To analyze the influence of intestinal flora on AFD, 76 chickens with different amounts of abdominal fat were selected for subsequent 16S rRNA gene studies. The 16S rRNA gene sequencing analysis produced a total of 7,745,067 quality-filtered effective tags from these samples, and 2,023 OTUs were then identified. The average Good’s-Coverage index for each sample was 0.993 (0.991–0.994), implying sufficient sequencing depth (Supplementary Figure 2).

All sequenced samples were divided into two groups based on the AFP, namely, HH and LL chickens. The AFD, including AFW and AFP, were significantly divergent between LL and HH chickens (both *P* = 0.000), and the AFD in the HH group was about twice that in the LL group (Supplementary Table 2). The carcass traits, such as BW, CW, and EW, were significantly different between the HH and LL groups (*P* < 0.05), but there was only a 1.05-times change for HH to LL chickens (Supplementary Table 2). None of the pectoralis lipid contents were significantly different between the two groups (Supplementary Table 2).

**FIGURE 2:**
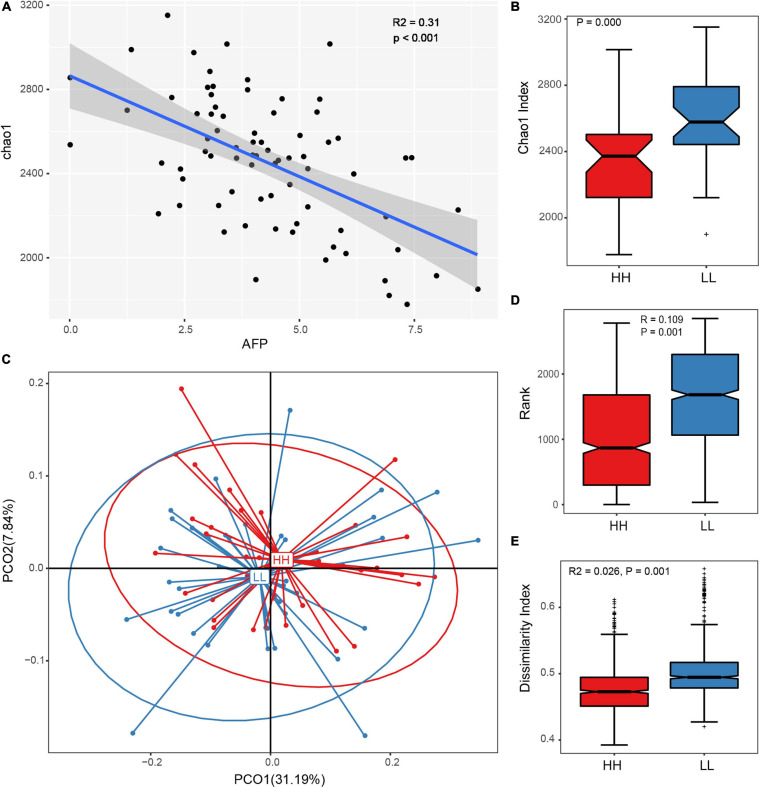
Gut microbial diversity and community with different abdominal fat deposition. **(A)** The Spearman’s correlation coefficient of the Chao1 index and the AFP of Tiannong Partridge Chickens, each dot represents on samples. **(B)** The comparison of Chao1 index between the high and low AFP chickens. **(C)** Represents the result of PCoA analysis based on OTUs of chickens with variant AF. **(D,E)** The results of Anosim and Adonis analyses. HH, high AFP chickens; LL, low AFP chickens.

The Spearman’s correlation coefficient of gut microbiota diversity and AFP suggested that the richness and diversity of the gut microbiota decreased with an increase in AFD (Figure 2A). The difference in microbial flora structure between the LL and HH groups further confirmed the close relationship between the gut microbiome and AFD of Tiannong Partridge Chickens. The alpha diversity suggested that AFD had significant effects on the gut microbiome. Specifically, the Shannon and Simpson indices of the two groups were not significantly different (Supplementary Figures 3A, 2B), but the Chao1 (Figure 2B), sobs (Supplementary Figure 2B), and ACE (Supplementary Figure 2C) indices of the HH group were all lower than those of the LL group (all *P* = 0.000). Furthermore, beta diversity analyses revealed different gut microbial communities among chickens with different levels of AFD. Even though no distinct separation was observed between the leaner and fatter chickens using PCoA (Figure 2C), the Anosim and Adonis analyses (Figures 2D,E) demonstrated greater inter-group diversity than inner-group diversity between the LL and HH groups (*P* = 0.001), implying a different gut microbial composition between Tiannong Partridge Chickens with different levels of AFD.

### Gut Microbes Associated With Abdominal Fat Deposition

A total of 1577 and 1515 OTUs were identified in the LL and HH groups, respectively, and 1375 of them were shared between the two groups. Subsequently, the OTUs were classified into 26 phyla, 64 classes, 97 orders, 164 families, and 332 genera.

**FIGURE 3:**
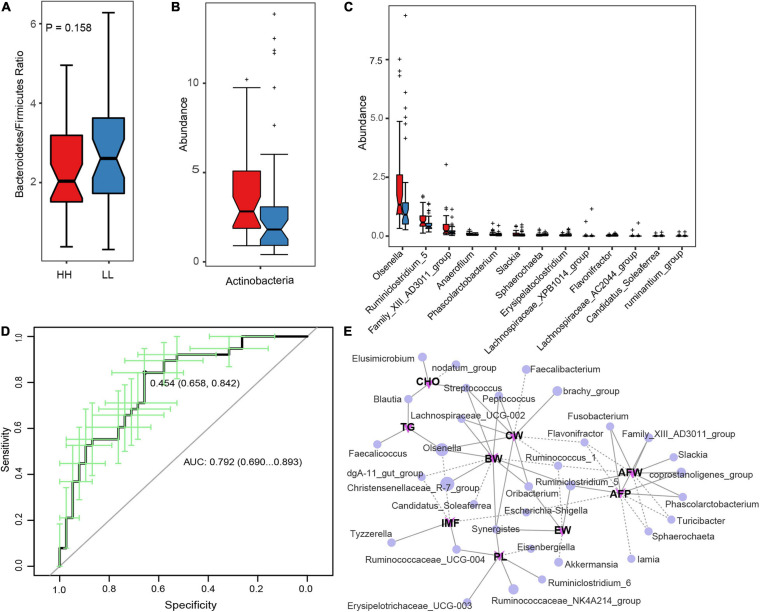
Correlation between gut microbes and abdominal fat deposition. **(A)** The Bacteroidetes/Firmicutes ratio between the HH and LL chickens. **(B,C)** The differentially enriched microbial phylum and genera, respectively. **(D)** Represents the AUC of genus microbiota based on the AFP classification. **(E)** The Spearman’s correlation network between genus microbiota and host phenotypes, the full line represents significant positive correlation (*P* < 0.05) while the dotted line represents significant negative correlation (*P* < 0.05). HH, high AFP chickens; LL, low AFP chickens. BW, body weight; CW, carcass weight; EW, eviscerated weight; AFW, abdominal fat weight; AFP, abdominal fat percentage; IMF, intramuscular fat; TG, triglyceride; PL, phospholipid; CHO, cholesterol.

To identify the microbes associated with the AFD in Tiannong Partridge chickens, the relative abundance of microbes was compared between LL and HH chickens. At phylum level, Bacteroidetes and Firmicutes dominated the gut microbial communities in both LL and HH chickens (Supplementary Figure 4A). However, there was no significant difference in the Bacteroidetes/Firmicutes ratio between the two groups (Figure 3A). Although some of the top 10 most abundant phyla had relatively great variation between the HH and LL groups (Supplementary Figure 4B), only Actinobacteria was more abundant in the HH chickens than in the LL chickens (P < 0.05) (Figure 3B). The relative abundances and the comparison of the top 10 abundant genera between the HH and LL groups were shown in Supplementary Figures 4C,D. Furthermore, the multi-test analysis revealed a total of 13 differentially enriched genera between the two groups (Figure 3C). Consistent with the results of the phylum comparison, the genera, *Olsenella* and *Slackia*, belonging to the phylum Actinobacteria were more enriched in HH chickens. The genus, *Sphaerochaeta*, belonging to phylum *Spirochaetae*, was the most significantly enriched in the LL chickens. The remaining ten differentially enriched genera were all classified as phylum Firmicutes, of which genera *Anaerofilum*, *Ruminiclostridium 5*, *Family XIII AD3011* group, and *Phascolarctobacterium* were more abundant in the HH group, while *Lachnospiraceae XPB1014* group, *Lachnospiraceae AC2044* group, *Flavonifractor*, *Candidatus Soleaferrea*, *Erysipelatoclostridium*, and *ruminantium* group were more abundant in the LL group.

Furthermore, a random forest classifier based on the microbial genus was constructed to evaluate the diagnostic value of the AFP-associated microbiome. As a result, 14 genera, containing all 13 differentially enriched genera, were complied with an area under the receiver operating curve (AUC) of 79.2% (Figure 3D), suggesting that the gut microbiota genera were distinguished between the HH and LL chickens. Among them, the ten most indicative genera were *Sphaerochaeta*, *Anaerofilum*, *Erysipelatoclostridium*, *Family XIII AD3011* group, *Ruminiclostridium 5*, *Flavonifractor*, *Slackia*, *Candidatus Soleaferrea*, *Olsenella*, and *Phascolarctobacterium* (Supplementary Figure 5). Figure 3E also illustrated the multiple positive actions of the genera *Ruminiclostridium* 5 and the negative actions of the genera *Flavonifractor* on chicken body growth and AFD. Meanwhile, the genera *Family XIII AD3011* group, *Ruminiclostridium 5*, *Slackia*, *Fusobacterium*, and *Phascolarctobacterium* might positively contribute to the AFD of Tiannong Partridge chickens. Network analysis revealed that the genus *Olsenella* was negatively associated with chicken pectoralis TG content and positively associated with BW and CW. The genera *Candidatus Soleaferrea* was also negatively associated with chicken BW. However, it also illustrated the multiple positive actions of the genera *Ruminiclostridium 5* and the negative actions of the genera *Flavonifractor* on chicken body growth and AFD. Additionally, the direct correlation analysis between the 13 differentially enriched genera and the host phenotype was completely consistent with the above results (Supplementary Figure 6).

### Shotgun Metagenomic Species Associated With Extreme Abdominal Fat Deposition Traits

To delve into the specific gut species associated with abdominal fat deposition, shotgun metagenomic sequencing analysis was performed on a subset of eight samples of LL and HH. These samples represent 4.58- and 3.47-times diversity between LL and HH for AFW and AFP, respectively (Supplementary Table 3). A total of 30,099,632-46,034,303 clean reads were generated for each sample. In total, 2,729,686 genes were annotated for these samples. Subsequently, 39 phyla, 132 classes, 332 orders, 787 families, 1,772 genera, and 5,542 species were obtained and compared between the LL and HH chickens. The co-occurrence microbiota classification phylogenetic tree suggested good consistency of the shotgun metagenomic and 16S rRNA gene sequencing results (Supplementary Figure 7).

**FIGURE 4:**
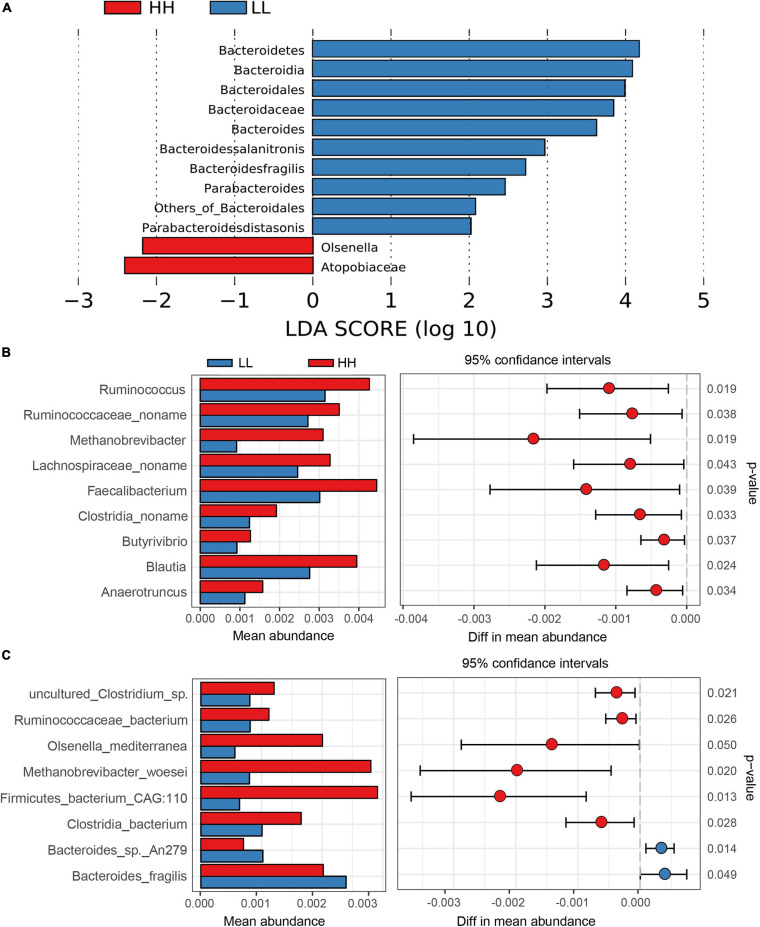
The taxonomic differences between high and low AFP chickens. **(A)** Differentially enriched gut microorganism identified by the LEfSe analysis of the taxonomic profiling based on the clean reads. **(B)** The differentially enriched gut microorganism genus based on gene annotation. **(C)** The differentially enriched gut microorganism species based on gene annotation. HH, high AFP chickens; LL, low AFP chickens.

The greater inter-group diversity than the inner-group diversity (*P* < 0.05) of the shotgun metagenomic microbiome demonstrated distinct shotgun metagenomic species composition between chickens with extreme AFD traits (Supplementary Figure 8). LEfSe analysis of the taxonomic profiling based on the clean reads was first performed to identify the different shotgun metagenomic species between the high and low AFP chickens. The results clearly showed that the phylum Bacteroidetes, genus *Bacteroides*, *Parabacteroides*, and *Olsenella*, species *Bacteroides salanitronis* (*B. salanitronis*), *Bacteroides fragilis* (*B. fragilis*), and *Parabacteroides distasonis* (*P. distasonis*) were differentially enriched in the LL versus HH groups (Figure 4A). Specifically, the percentage of Bacteroidetes was higher in the lean (19.61%) than in the fat chickens (16.18%). The subordinate genera *Bacteroides* and *Parabacteroides* were more enriched in the lean (12.10% and 0.80%) than in the fat chickens (9.29% and 0.65%, respectively). Accordingly, *B. salanitronis* and *B. fragilis* as well as *P. distasonis*, were also more abundant in the lean (4.11%, 3.63%, and 0.80%) than in the fat chickens (2.79%, 2.98%, and 0.65%), respectively. Conversely, the percentage of genus *Olsenella* was higher in the fat (0.15%) than in the lean line (0.06%).

In addition, taxonomic differences at genus and species levels were identified based on the results of gene annotation between the HH and LL groups. As a result, phylum Bacteroidetes was notably more enriched in the LL group, while phylum Euryarchaeota was more enriched in the HH group. Nine genera were found to be significantly different between the HH and LL chickens, and all of them were more abundant in the HH group (Figure 4B). In order of significance, the different enriched genera included *Ruminococcus*, *Methanobrevibacter*, *Blautia*, *Clostridia noname*, *Anaerotruncus*, *Butyrivibrio*, *Ruminococcaceae noname*, *Faecalibacterium*, and *Lachnospiraceae noname*. In addition, a total of eight differentially enriched species were identified between the HH and LL groups (Figure 4C). Among them, *B. fragilis* and *B. sp. An279* were more abundant in the LL chickens, whereas the remaining six species, *Firmicutes bacterium CAG:110*, *Methanobrevibacter woesei* (*M. woesei*), *Olsenella mediterranea* (*O. mediterranea*), *Clostridia bacterium* (*C. bacterium*), *Ruminococcaceae bacterium* (*R. bacterium*), and *uncultured Clostridium sp.* were more abundant in the HH chickens.

### Alterations of Microbial Function Associating to Abdominal Fat Deposition

To illustrate the functional alterations within the gut microbiome between high and low AFP chickens, the shotgun metagenomic genes were annotated to KO modules and KEGG pathways. Most genes in both LL and HH groups were annotated to carbohydrate metabolism, followed by amino acid metabolism (Supplementary Figure 9). In terms of KEGG pathways, Anosim and Adonis analyses demonstrated greater inter-group than inner-group diversity between the LL and HH groups (*P* = 0.03). NMDS analysis suggested distinct microbial functions associated with different AFDs of Tiannong Partridge Chickens (Supplementary Figure 10). In detail, 19 pathways were annotated by the differentially expressed KO modules between the LL and HH groups, and all these pathways belonged to the functional classification of metabolism (Table 1). Among them, seven pathways were upregulated in the HH chickens, including “metabolic pathways,” “degradation of aromatic compounds,” “sphingolipid metabolism,” “galactose metabolism,” “methane metabolism,” “oxidative phosphorylation,” and “phenylalanine, tyrosine, and tryptophan biosynthesis.” Among them, six pathways belonged to the functional classes of “global and overview maps,” “lipid metabolism,” “carbohydrate metabolism,” and “energy metabolism.” Six pathways, including “photosynthesis,” “glycine, serine, and threonine metabolism,” “cyanoamino acid metabolism,” “riboflavin metabolism,” “monobactam biosynthesis,” and “dioxin degradation” were downregulated in the HH chickens. These pathways are associated with the metabolism of amino acids, cofactors, secondary metabolites, and xenobiotics. In addition, six pathways, mainly associated with the metabolism of glycan, amino acids, cofactors, and vitamins, were annotated by both overexpressed and downregulated genes, including “lipopolysaccharide biosynthesis,” “other glycan degradation,” “phosphonate and phosphinate metabolism,” “biotin metabolism,” “folate biosynthesis,” and “ubiquinone and other terpenoid-quinone biosynthesis.”

**TABLE 1:** The regulation of microbial function in the high AFP chickens comparing to the low AFP chickens.

Pathway ID	KEGG pathway class	Pathway	Down expression genes	Up expression genes
ko01100	Global and overview maps	Metabolic pathways	–	307
ko01220	Global and overview maps	Degradation of aromatic compounds	–	5
ko00600	Lipid metabolism	Sphingolipid metabolism	–	10
ko00052	Carbohydrate metabolism	Galactose metabolism	–	23
ko00680	Energy metabolism	Methane metabolism	–	24
ko00190	Energy metabolism	Oxidative phosphorylation	–	20
ko00400	Amino acid metabolism	Phenylalanine, tyrosine and tryptophan biosynthesis	–	18
ko00195	Energy metabolism	Photosynthesis	1	–
ko00260	Amino acid metabolism	Glycine, serine and threonine metabolism	12	–
ko00460	Metabolism of other amino acids	Cyanoamino acid metabolism	6	–
ko00740	Metabolism of cofactors and vitamins	Riboflavin metabolism	4	–
ko00261	Biosynthesis of other secondary metabolites	Monobactam biosynthesis	5	–
ko00621	Xenobiotics biodegradation and metabolism	Dioxin degradation	1	–
ko00540	Glycan biosynthesis and metabolism	Lipopolysaccharide biosynthesis	6	8
ko00511	Glycan biosynthesis and metabolism	Other glycan degradation	8	19
ko00440	Metabolism of other amino acids	Phosphonate and phosphinate metabolism	3	4
ko00780	Metabolism of cofactors and vitamins	Biotin metabolism	6	10
ko00790	Metabolism of cofactors and vitamins	Folate biosynthesis	7	11
ko00130	Metabolism of cofactors and vitamins	Ubiquinone and other terpenoid-quinone biosynthesis	4	5

Furthermore, the shotgun metagenomic genes were annotated to the eggNOG and CAZy databases. The Anosim and Adonis analyses suggested different microbial functions in both eggNOG (*P* = 0.04) and CAZy (*P* = 0.03) for the gut microbiota in LL and HH chickens, which were further evidenced by the NMDS analyses (Supplementary Figures 11, 12). Specifically, 200 orthologous groups (OG) were enriched, and 19 of them were differentially expressed between the HH and LL chickens (Supplementary Table 4). Among them, only three OGs, representing cell wall/membrane/envelope biogenesis and transcription, were high in HH chickens. The remaining OGs were more highly expressed in the LL chickens, suggesting more active microbial functions of carbohydrate transport and metabolism, energy production and conversion, and inorganic ion transport and metabolism in the LL chickens. However, 326 CAZy enzymes belonging to 6 CAZy activities were enriched. Most shotgun metagenomic genes of Tiannong Partridge Chickens were enriched in glycoside hydrolases (GH), glycosyltransferases (GT), and carbohydrate-binding modules (CBM) (Supplementary Figure 13). The HH and LL chickens had 25 different CAZy enzymes (Supplementary Table 5), of which 9 enzymes had high expression in HH group, while 16 enzymes were highly expressed in the LL groups. Four carbohydrate-binding modules (CBM), including family 50 (CBM50), 13 (CBM13), 34 (CBM34), and 37 (CBM37), were all high in the HH chickens. Glycoside hydrolase family 42 (GH42) and 49 (GH49), as well as glycosyltransferase family 39 (GT39), 66 (GT66), and 7 (GT7), were also highly expressed in the HH chickens. Meanwhile, the two polysaccharide lyases (PL0 and PL33) and the two carbohydrate esterases (CE2 and CE6) showed high expression in the LL chickens. In addition, a total of nine glycoside hydrolases (GH10, GH109, GH11, GH146, GH16, GH29, GH30, GH35, and GH67) and three glycosyltransferases (GT11, GT3, and GT30) were also highly expressed in the LL chickens.

### Co-occurrence Network of Microbial Taxa and Function Capacities With Phenotypic Traits

**FIGURE 5:**
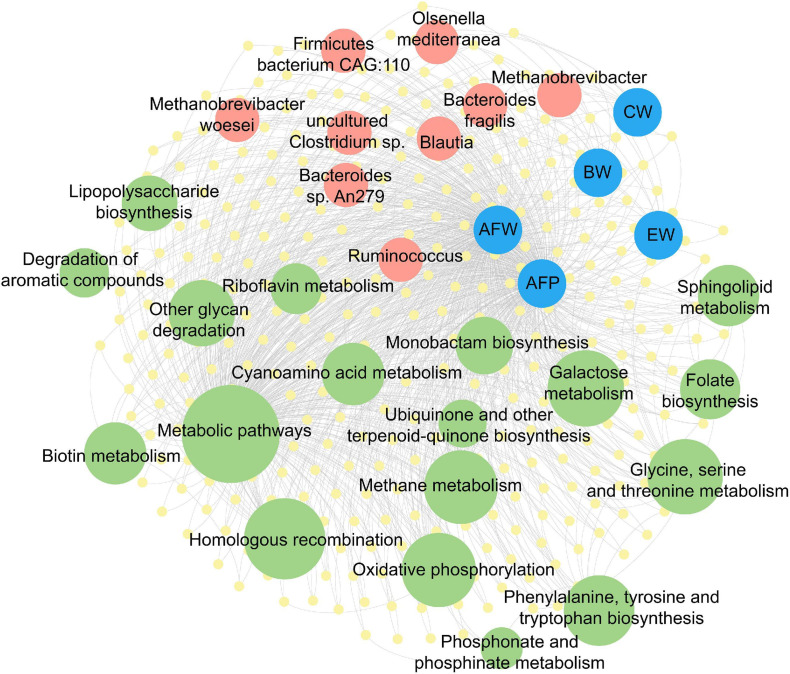
Co-occurrence network of microbial taxa and function capacities with chicken phenotypic traits. The taxa (Red circles) and pathways (green circles) were annotated based on the (yellow circles). The yellow circles represent the differentially expressed genes, red circles represent the differentially enriched taxa, green circles in different size represent the enriched pathways with different degrees of enrichment, while blue circles represent the related chicken phenotypic traits. The gray lines represent edges with Pearson’s correlation coefficient > 0.8 or < −0.8 and *P* < 0.05. BW, body weight; CW, carcass weight; EW, eviscerated weight; AFW, abdominal fat weight; AFP, abdominal fat percentage.

To understand the contribution of the gut microbiota in chicken fat accumulation, a co-occurrence network representing microbial interactions of differentiated microbial taxa function capacities with phenotypic traits was constructed (Figure 5 and Supplementary Table 6). Traits of AFW and AFP and carcass traits (BW, CW, and EW) had strong correlation with gut microbial community (Pearson’s correlation coefficient > 0.8 or < −0.8, and *P* < 0.05) while the pectoralis lipid composition related traits (IMF, TG, CHO, and PL) were weakly correlated to the gut microbiome. Nine microbial taxa including three genera (*Methanobrevibacter*, *Ruminococcus*, and *Blautia*) and six species (*M. woesei*, *O. mediterranea*, *B. fragilis*, *B. sp. An279*, *Firmicutes bacterium CAG:110*, and *uncultured Clostridium sp*.) based on differentially expressed genes between the lean and fat chickens were enriched to several lipid and carbohydrate metabolism pathways. The most enriched pathways included “metabolism pathways,” “methane metabolism,” “lipopolysaccharide biosynthesis,” “other glycan degradatin,” “phenylalanine, tyrosine and tryptophan biosynthesis,” “oxidative phosphorylation,” “Glycine, serine and threonine metabolism,” and “galactose metabolism”. Notably, genera *Methanobrevibacter* and its subordinate *M. woesei* and *O. mediterranea* were shown participating to pathways including “metabolism pathways,” “methane metabolism,” and “glycan degradatin” and were strongly correlated to all AFD and carcass traits. *Firmicutes bacterium CAG:110* had close relationship with both AFW and AFP. *B. fragilis* and *B. sp. An279* only had close relationship with AFW but not AFP, while *Blautia* had strong correlation just with chicken carcass traits.

## Discussion

As the commercial line of the Chinese local chicken breed Qingyuan Partridge Chickens and Guangxi Partridge Chickens, Tiannong Partridge chickens completely retains the high quality of meat and flavor and has been selected for automatic sexing, early maturity, and better growth performance. By determining the AFD, carcass traits, and pectoralis lipid compositions of a random flock, this study revealed the differences in AFD among Tiannong Partridge Chickens. Like those in previous reports (Jiang et al., 2017; Abdalla et al., 2018), our results show that chickens genetically selected for earlier maturation and faster growth are characterized by increased AFD. The great variation and normal distribution of AFW and AFP in Tiannong Partridge Chickens suggest that this population is representative for the studying the contribution of gut microbes to AFD in the chicken industry. Meanwhile, the inconsistency between AFD and carcass traits also indicates that it is possible to manipulate the deposition of chicken abdominal fat while maintaining carcass traits (Jiang et al., 2017).

With the global investigation on gut microbial abundance related to AFD, our dataset shows that the richness and diversity of gut microbiota decrease along with an increase in chicken AFD, and that the gut microbial community is greatly affected by different levels of AFD. These results are in line with those of previous studies in humans (Li R. et al., 2020), mice (Ellekilde et al., 2014; Kong et al., 2019), and pigs (Qi et al., 2019). In this study, the comparison of the abundance of microbial taxa reveals that several microbes could be markers of the various levels of AFD. Although the Bacteroidetes/Firmicutes ratio in the HH and LL chickens is not significantly different based on the 16S analysis, a reduction in the abundance of Bacteroidetes and a proportional increase in Firmicutes are observed in HH chickens. Shotgun metagenomic analysis of the extremely high and low AFP chickens also shows less Bacteroidetes abundance in the fatter chickens. As two of the most abundant and ubiquitous chicken gut microbiota taxa, Firmicutes and Bacteroidetes have been widely reported to have varying numbers of relative abundances in fat and lean mice, and obesity has been correlated with a shift in the abundance of both Bacteroidetes and Firmicutes (Ley et al., 2005, 2006). Moreover, previous studies have shown that a high-fat diet changes the relative composition of the gut microbiota by increasing Firmicutes and decreasing Bacteroidetes at the phylum levels in both humans and mice (Serino et al., 2012; David et al., 2014; Zheng et al., 2017).

Different levels of selection-acquired obesity not only alters the composition of the gut microbiota, but also influences their functional performance by enriching their relative abundance in microbial taxa. One mechanism through which the gut microbiota contribute to fat deposition is by providing microbial metabolic pathways that are not encoded by the host genome, and thus, regulating the host nutritional metabolism, including nutrient integration and energy capture (Hou et al., 2016; Schmidt et al., 2018).

Our results show that the Bacteroidetes phylum is closely associated with chicken AFD and its subordinate genera *Bacteroides* and *Parabacteroides* are more enriched in the lean chickens. Accordingly, the species *B. salanitronis*, *B. fragilis*, and *P. distasonis*, are less abundant in fat chickens. A previous study has revealed that Bacteroidetes are involved in many metabolic activities, including fermentation of carbohydrates, utilization of nitrogenous substances, and biotransformation of bile acids and steroids (Lan et al., 2006). In this study, the microbiota of LL chickens had high CAZy enzyme activities in carbohydrate esterase, polysaccharide lyase, and glycoside hydrolase for glucans and galactans as well as glycosyltransferase for nucleotide monophosphosugar. Furthermore, orthologous groups related to carbohydrate and inorganic ion transport and metabolism, as well as high energy production and conversion, show high abundance in the LL chicken gut microbiome. Moreover, pathways associated with the metabolism of amino acids, cofactors, secondary metabolites, and xenobiotics are found to be more enriched in LL chickens. These functional alternations are closely related to the Bacteroidetes phylum, which is further supported by correlation analyses of host phenotypes to different gut microorganisms. As it has been reported (Gronow et al., 2011), *B. salanitronis* contributes to the breakdown of food, produces nutrients and energy needed by the chicken, and can ferment glucose, sucrose, arabinose, cellobiose, lactose, xylose, and raffinose. However, it does not utilize trehalose, glycerol, mannitol, sorbitol, or melezitose (Gronow et al., 2011). On the other hand, the presence of phosphoenolpyruvate-oxaloacetate catalytic enzymes gene in *B. fragilis* genome may indicate the potential for efficient propionate synthesis (Xu et al., 2020). In the long-term evolutionary process, *B. fragilis* colonizes the host intestine, participates in the fermentation of glucose, fructose, galactose, lactose, sucrose, dextrin, etc., and plays an important role, especially in obesity, diabetes, and immunodeficiency diseases (Zhao et al., 2019; Donaldson et al., 2020). Meanwhile, *P. distasonis* has also been reported modulating host metabolism and alleviating obesity and metabolic dysfunctions via the production of succinate and secondary bile acids (Wang et al., 2019).

On the other hand, the subordinate genera of phylum Firmicutes, *Phascolarctobacterium*, *Family XIII AD3011* group, and *Ruminiclostridium 5*, are found to be more abundant in the high AFP chickens using 16S analyses. Moreover, eight of the nine differentially enriched genera between the extremely high and low AFP chickens, including *Anaerotruncus*, *Blautia*, *Butyrivibrio*, *Clostridia noname*, *Faecalibacterium*, *Lachnospiraceae noname*, *Ruminococcaceae noname*, and *Ruminococcus*, belong to the phylum Firmicutes and all are more abundant in the HH chickens. *Phascolarctobacterium* has been suggested associating with host metabolic state and mood by producing short-chain fatty acids (SCFA) such as acetate and propionate (Wu et al., 2017). Meanwhile, taxa of two predominantly butyrate-producing genus, *Faecalibacterium* and *Ruminococcus*, are also reported significantly more prevalent in obese individuals than in non-obese individuals (Maniar et al., 2019). As shown in the cecum and colon of rats (Shi et al., 2020), the increase in the *Family XIII AD3011* group may be involved in the production of skatole and indole. Moreover, the families *Lachnospiraceae*, *Clostridia*, and *Ruminococcaceae*, and genera *Ruminiclostridium*, *Blautia*, and *Butyrivibrio*, have been suggested as high fat diet-dependent gut taxa and are likely associated with lipid metabolism (Lin et al., 2016; Zietak et al., 2016; Kong et al., 2019; Hou et al., 2020). Specifically, the butyrate producing *Lachnospiraceae* and *Ruminococcaceae* are suggested to reduce lipopolysaccharide biosynthesis in mice (Kang et al., 2017). Therefore, it is reasonable for the both up- and down-regulation of lipopolysaccharide biosynthesis in the HH chickens, by considering the more abundant of *Lachnospiraceae noname* and less abundant of *Lachnospiraceae XPB1014* group and *Lachnospiraceae AC2044* group in the high AFP chickens. And according to the C_2_–C_18_ fatty acid tests, *Clostridium perfringens* has the highest activity toward lauric acid (Kang et al., 2017). Owing to the widespread existence of these taxa, the microbiota in the HH chickens have more activities of carbohydrate-binding modules and high functional expression of lipids, carbohydrates, and energy metabolism. This is in line with the results that the increase in circulating lipoprotein lipase activity caused by gut microbiota results, in turn, in a significant increase in body fat deposition in the host.

Moreover, a high abundance of phylum Actinobacteria and its subordinate genera, *Olsenella* and *Slackia*, are observed in the HH chickens. A high abundance of *Olsenella* and *Slackia* has been observed in mice fed a high-fat diet (Gohir et al., 2019; Nagpal et al., 2019), suggesting their strong correlation with fat and energy metabolism. *Olsenella* has been suggested to positively correlate to methane metabolism and contribute to the metabolic pathways of glycolysis/gluconeogenesis, carbon fixation in photosynthetic organisms, pentose phosphate pathway, and ascorbate and aldarate metabolism (Zhang Y. et al., 2020). This may provide a potential mechanism for the positive correlation between the abundance of genus *Olsenella* to chicken BW, CW, and TG. In addition, phylum Euryarchaeota and its subordinate genus *Methanobrevibacter* and species *M. woesei* were also more abundant in the high AFP chickens. *Methanobrevibacter* is a common and important methanogenic taxon primarily inhabiting the cecum of chickens. Chickens with fewer *Methanobrevibacter* have significantly lower abdominal fat content than those with a higher abundance of *Methanobrevibacter* (35.51 vs. 55.59 g, respectively) (Wen et al., 2019). Apart from bacteria, the dominant gut species, *Methanobrevibacter smithii*, has been found extensively colonizing the small bowel as well as colon, and affects host calorie harvest and adiposity through the digestion of dietary polysaccharides (Hansen et al., 2011; Mathur et al., 2013). This subsequently improves the efficiency of microbial fermentation and enhances host energy capture. In addition, *Methanobrevibacter* has been suggested to improve acetate and butyrate production and eliminate hydrogen and formate, which are vital carbon sources for colon epithelial cells (Samuel et al., 2007; Hansen et al., 2011). The increase in lipoprotein lipase activity in the villi of epithelia caused by gut microbiota leads to increased triglyceride uptake and peripheral fat storage. In this study, the methanogenic taxa, genus *Methanobrevibacter* and species *M. woesei*, were found to participate in the regulation of gut metabolism including methane metabolism, photosynthesis-antenna proteins, and various types of N-glycan biosynthesis. Our results also suggest a limited association of *Methanobrevibacter* abundance with other gut microbiota or any carcass traits, further supporting the feasibility of reducing fat deposition by inhibiting the caeca-associated genus, *Methanobrevibacter*, without affecting the proportion of carcass meat (Wen et al., 2019).

By revealing the strong correlations between the identified bacterial groups and the phenotypes related to chicken abdominal fat deposition, the present study demonstrates that gut microbiota is an important factor involving AFD in conventional chicken breeds. Moreover, some of the observed differential taxa and potential genes/metabolic pathways are suggested as possible biomarkers associated with chicken AFD. However, we acknowledge that the current dataset lacks evidence supporting the cause-effect relationship of specific taxon to AFD, since it does not provide what microbes are doing or the metabolites that they produce. Study using chickens involving administration of the candidate microbes are needed to further validate the contribution of these microbiota to chicken abdominal fat deposition. Furthermore, studies on the real expression of the suggested genes and metabolic pathways are expected to investigate the mechanistic and functional connection with AFD.

## Conclusion

In conclusion, alterations in the gut microbiome and its association with metabolism capacity have preliminarily elucidated the contribution of gut microbiota to chicken abdominal fat deposition. The richness and diversity of the gut microbiota decrease as the accumulation of chicken abdominal fat increases. The decrease of Bacteroidetes and the increase of Firmicutes are correlated with the accumulation of chicken abdominal fat deposition. The Bacteroidetes phylum, including *Bacteroides*, *Parabacteroides*, and the species, *B. salanitronis*, *B. fragilis*, and *P. distasonis*, were correlated to alleviate obesity by producing secondary metabolites. Several genera of Firmicutes phylum with circulating lipoprotein lipase activity were linked to the accumulation of chicken body fat. Moreover, the genera, *Olsenella* and *Slackia*, might positively contribute to fat and energy metabolism, whereas the genus, *Methanobrevibacter*, was possible to enhance energy capture, and associated to accumulate chicken abdominal fat deposition. These findings provide insights into the roles of the gut microbiota in complex traits and contribute to the development of effective therapies for the reduction of chicken fat accumulation.

## Data Availability Statement

The raw data of 16S rRNA gene presented in the study are deposited in NCBI repository, accession number SRR13782987-SRR13783062, while the raw data of shotgun metagenomic sequencing are deposited in NCBI under accession number SRR13783083-SRR13783090.

## Ethics Statement

The animal study was reviewed and approved by Laboratory Animal Welfare and Animal Experimental Ethical Inspection board of Foshan University. Written informed consent was obtained from the owners for the participation of their animals in this study.

## Author Contributions

HL, GZ, and HX designed this project. HX, JG, DZ, JL, HZ, YY, ST, GL, CL, and ZX performed the experiments and analyzed the data. HX, GZ, and HL interpreted the data and drafted the manuscript. All authors contributed critically to the drafts and gave final approval for the publication.

## Conflict of Interest

JG, HY, and HL were employed by the company Guangdong Tinoo’s Foods Group Co., Ltd. The remaining authors declare that the research was conducted in the absence of any commercial or financial relationships that could be construed as a potential conflict of interest.

## Supplementary Material

Supplementary Figure 1 The statistic distribution of the abdominal fat deposition and the pectoralis lipid composition and their correlations. BW, body weight; CW, carcass weight; EW, eviscerated weight; AFW, abdominal fat weight; AFP, abdominal fat percentage; IMF, intramuscular fat; TG, triglyceride; PL, phospholipid; CHO, cholesterol. The value in the upper triangular matrix represents the correlation coefficient; one * represents *P* < 0.05, two * represents *P* < 0.01 and three * represents *P* < 0.001.

Supplementary Figure 2 Good’s-Coverage index for all samples.

Supplementary Figure 3 Alpha diversity comparison between HH and LL. **(A)** Shannon index. **(B)** Simpson index. **(C)** Sobs index. **(D)** ACE index. HH, high AFP chickens; LL, low AFP chickens.

Supplementary Figure 4 The top 10 abundant phyla and genera between HH and LL groups. **(A,B)** The microbial composition and comparison of the top 10 abundant phyla, respectively. **(C,D)** show the microbial composition and comparison of the top 10 abundant genera, respectively.

Supplementary Figure 5 Random forest analyses on the of LL and HH chickens.

Supplementary Figure 6 Correlation analyses between the differentially enriched genera and the host phenotype. BW, body weight; CW, carcass weight; EW, eviscerated weight; AFW, abdominal fat weight; AFP, abdominal fat percentage; IMF, intramuscular fat; TG, triglyceride; PL, phospholipid; CHO, cholesterol. The background color represents the correlation coefficient; one * represents *P* < 0.05 and two * represents *P* < 0.01.

Supplementary Figure 7 The consistency of the results of the shotgun metagenomic and 16S rRNA gene sequencing. The small dots represent species, and the evolutionary branch tree represents the Kingdom, Phylum, Class, Order, Family, Genus and Species from the inside to the outside accordingly. The microorganisms with blue background were identified by both shotgun metagenomic sequencing and 16S rRNA gene sequencing, the microorganisms with green background were identified only by shotgun metagenomic sequencing, while the microorganisms with red background were identified only by 16S rRNA gene sequencing. The dot size indicates the average abundance of the microorganisms in all samples.

Supplementary Figure 8 The Anosim and Adonis comparison on Phylum composition between chickens with extreme abdominal fat deposition traits. HH, high AFP chickens; LL, low AFP chickens.

Supplementary Figure 9 Number of genes annotated to KEGG pathways for all samples.

Supplementary Figure 10 NMDS analysis based on KEGG pathways. HH, high AFP chickens; LL, low AFP chickens.

Supplementary Figure 11 NMDS analysis for eggNOG.C annotation. HH, high AFP chickens; LL, low AFP chickens.

Supplementary Figure 12 NMDS analysis based on CAZy activities. HH, high AFP chickens; LL, low AFP chickens.

Supplementary Figure 13 Number of genes annotated to each CAZy activities for all samples.
