# Supplementary figures and images for: Specific Microbial Taxa and Functional Capacity Contribute to Chicken Abdominal Fat Deposition
Source: Front Microbiol. 2021 Mar 17;12:643025. doi: 10.3389/fmicb.2021.643025 (PMC8010200; doi:10.3389/fmicb.2021.643025)

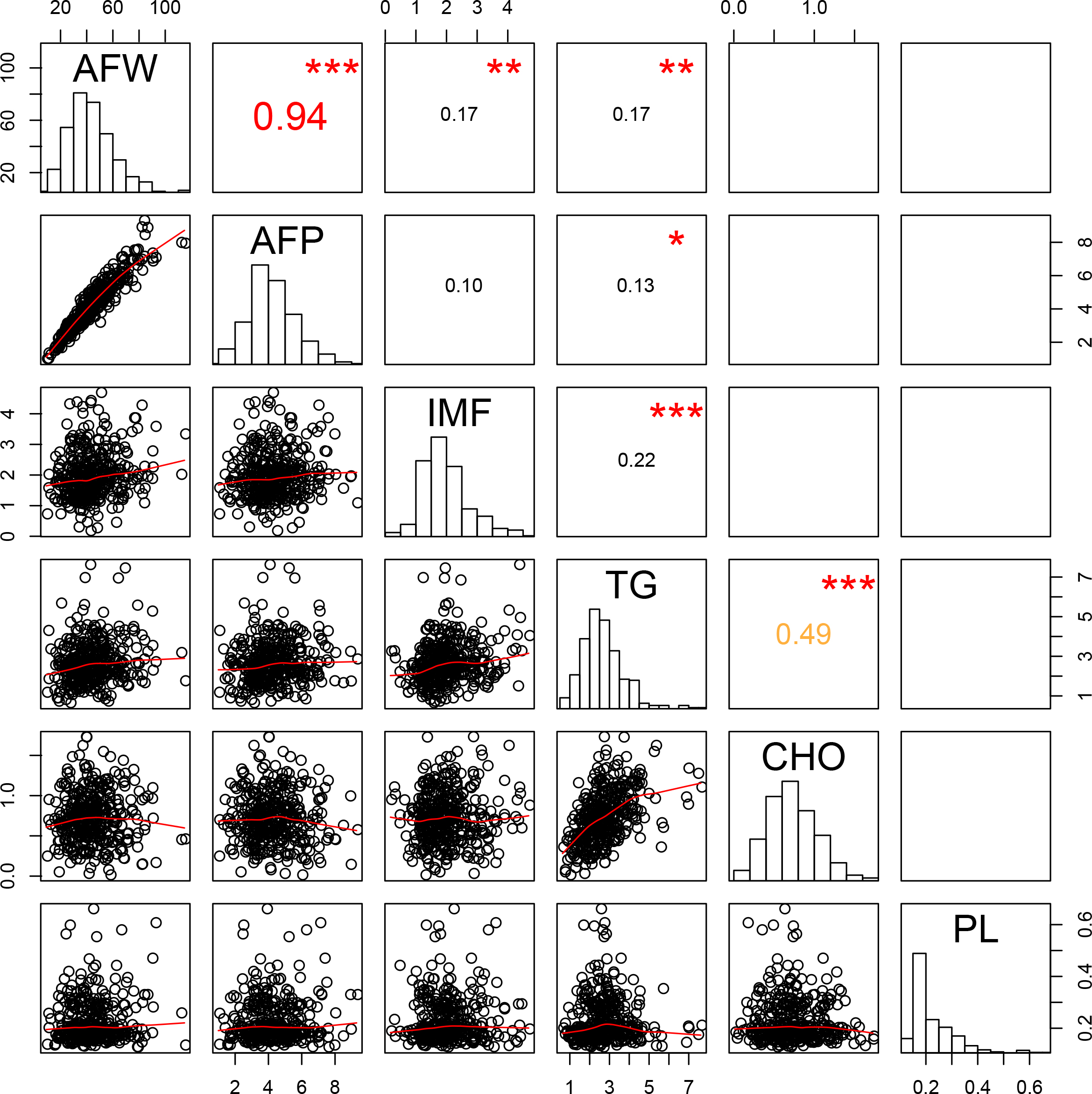

Supplement: Supplementary Figure 1 — The statistic distribution of the abdominal fat deposition and the pectoralis lipid composition and their correlations. BW, body weight; CW, carcass weight; EW, eviscerated weight; AFW, abdominal fat weight; AFP, abdominal fat percentage; IMF, intramuscular fat; TG, triglyceride; PL, phospholipid; CHO, cholesterol. The value in the upper triangular matrix represents the correlation coefficient; one * represents P < 0.05, two * represents P < 0.01 and three * represents P < 0.001. [file Image_1.JPEG]

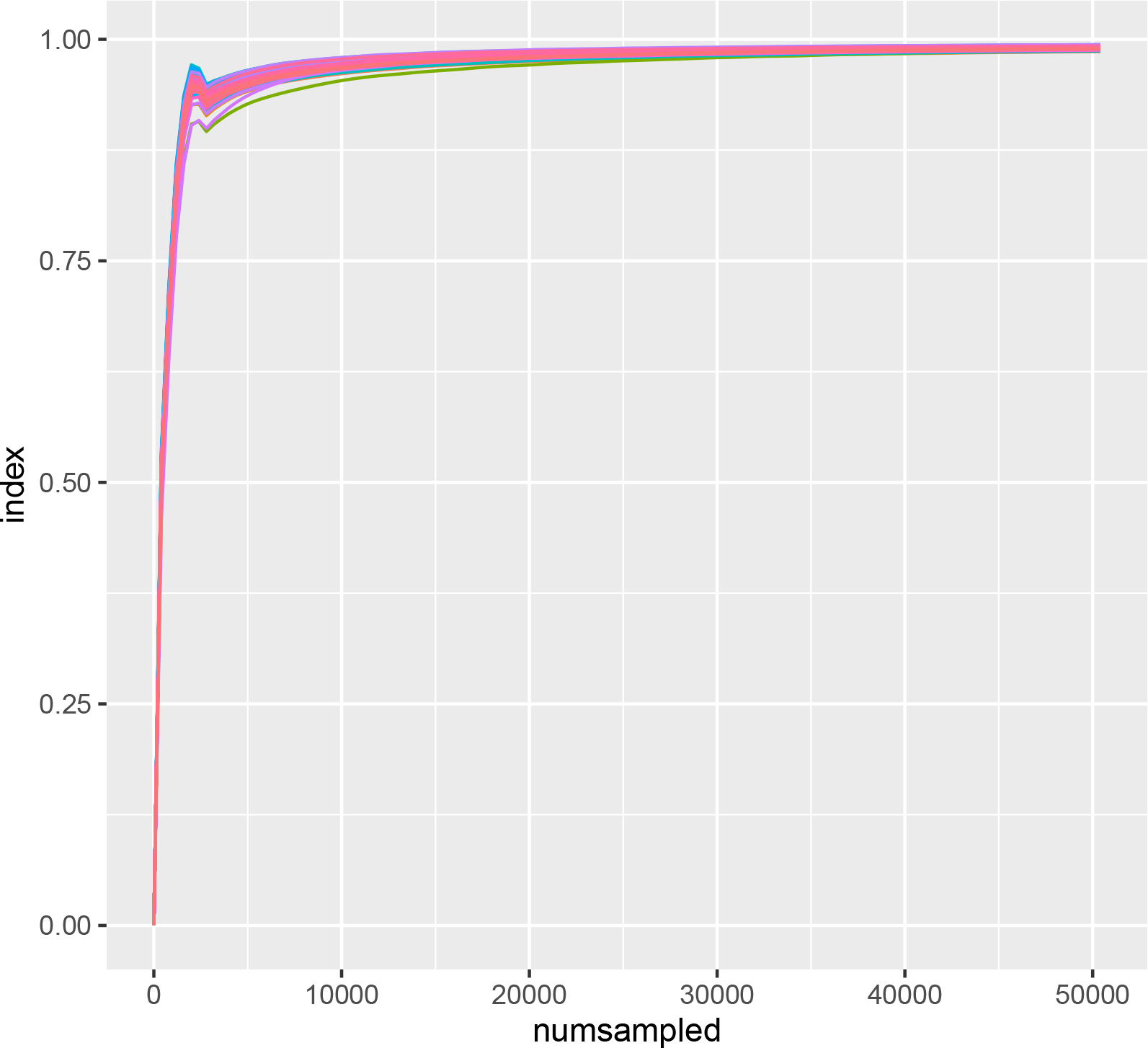

Supplement: Supplementary Figure 2 — Good’s-Coverage index for all samples. [file Image_2.JPEG]

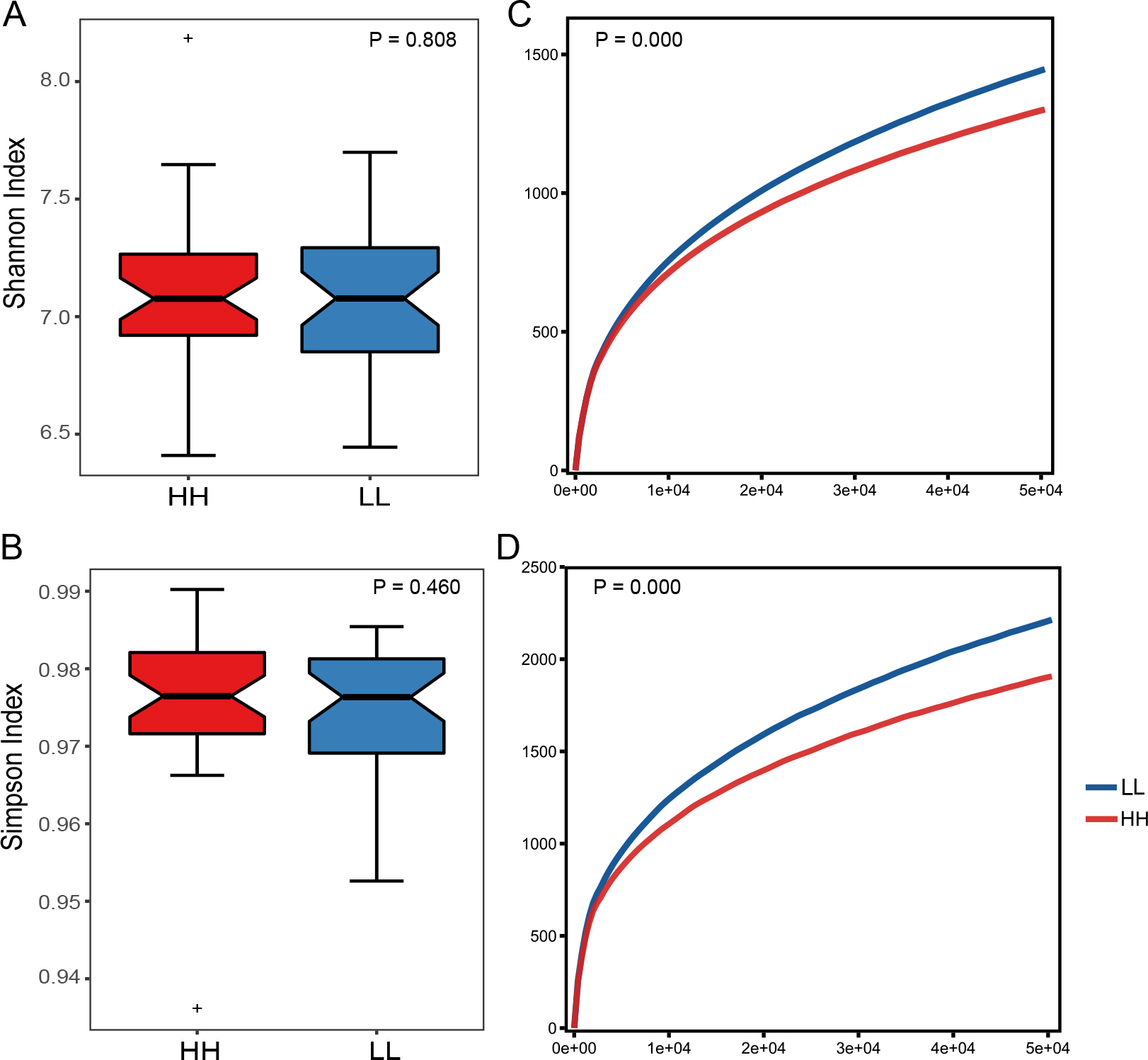

Supplement: Supplementary Figure 3 — Alpha diversity comparison between HH and LL. (A) Shannon index. (B) Simpson index. (C) Sobs index. (D) ACE index. HH, high AFP chickens; LL, low AFP chickens. [file Image_3.JPEG]

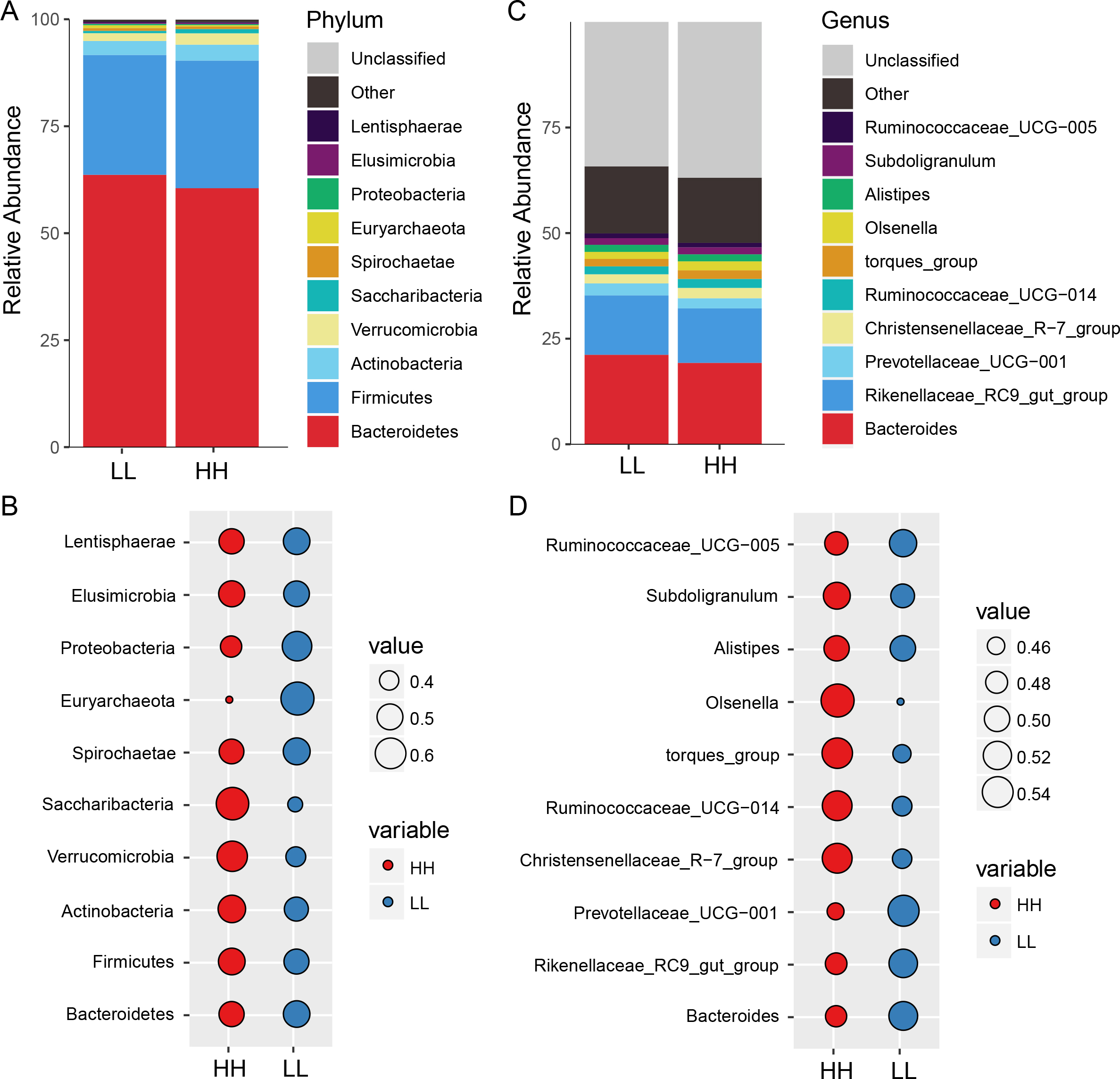

Supplement: Supplementary Figure 4 — The top 10 abundant phyla and genera between HH and LL groups. (A,B) The microbial composition and comparison of the top 10 abundant phyla, respectively. (C,D) show the microbial composition and comparison of the top 10 abundant genera, respectively. [file Image_4.JPEG]

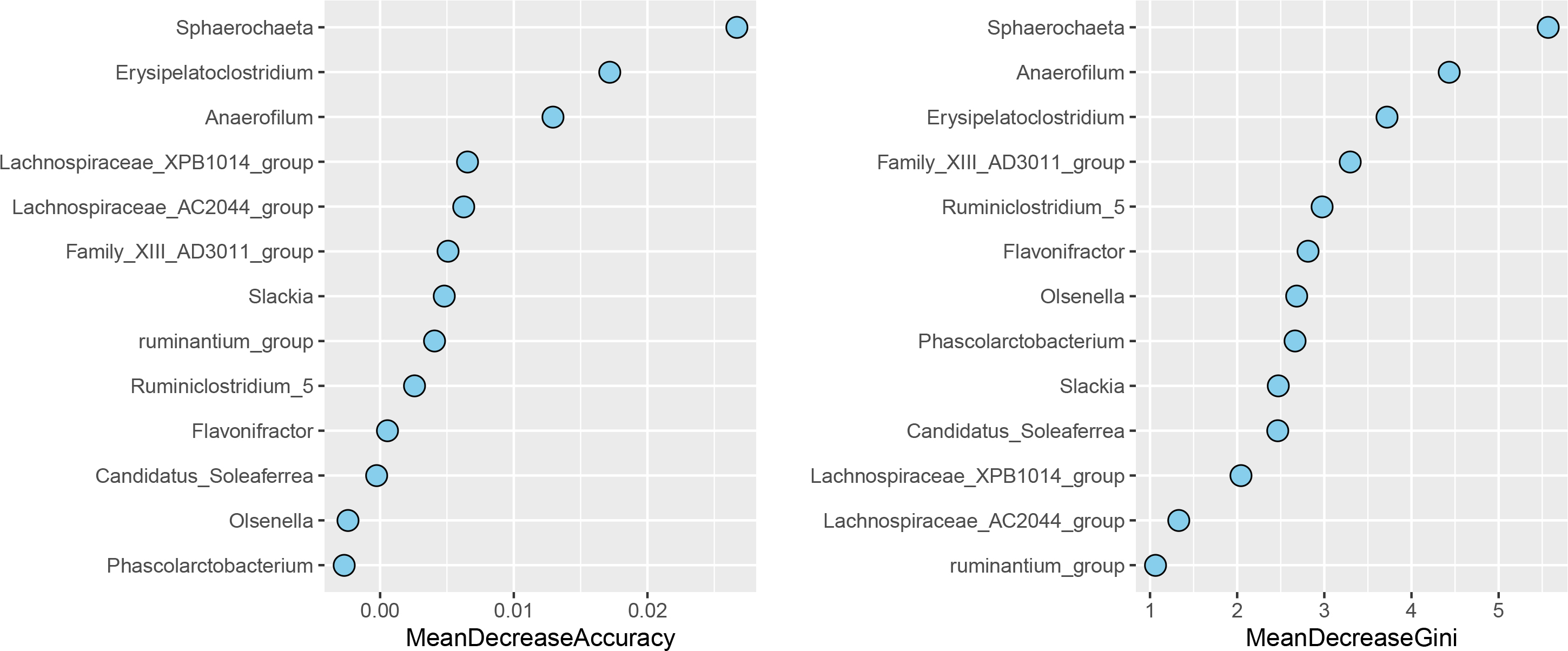

Supplement: Supplementary Figure 5 — Random forest analyses on the of LL and HH chickens. [file Image_5.JPEG]

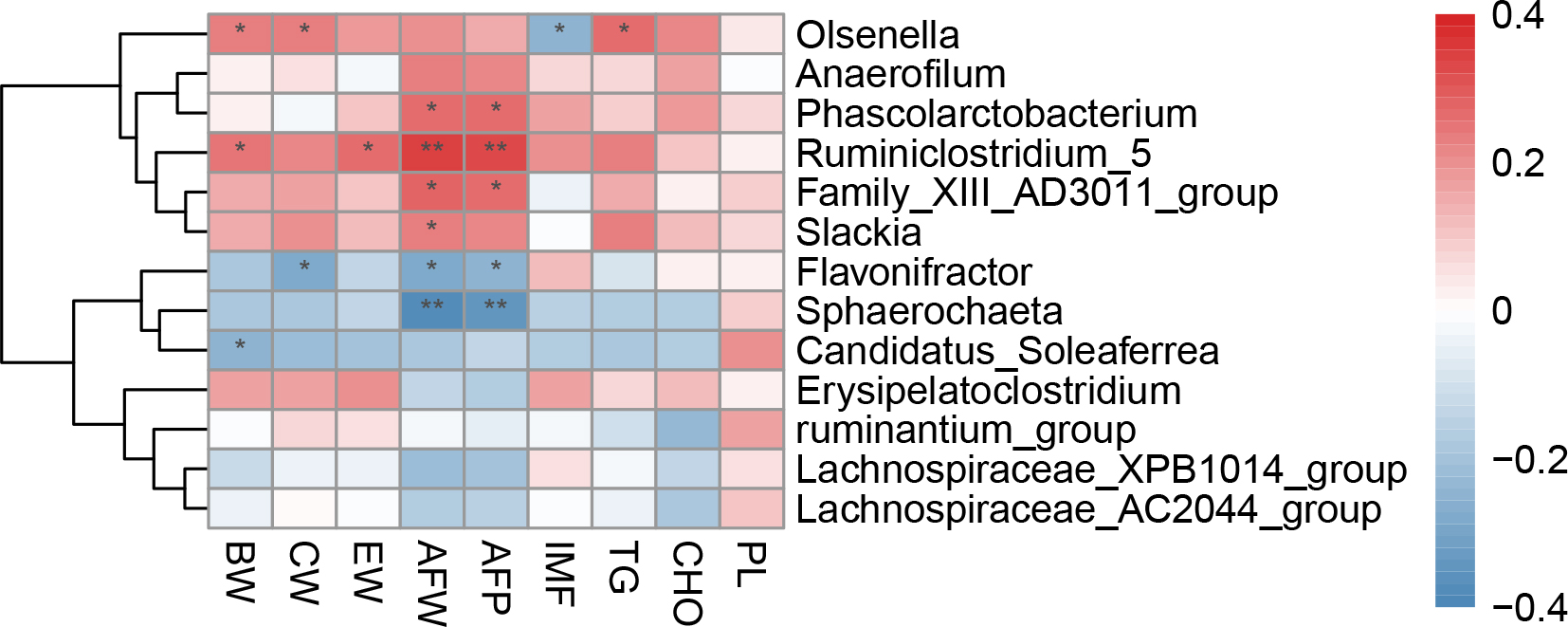

Supplement: Supplementary Figure 6 — Correlation analyses between the differentially enriched genera and the host phenotype. BW, body weight; CW, carcass weight; EW, eviscerated weight; AFW, abdominal fat weight; AFP, abdominal fat percentage; IMF, intramuscular fat; TG, triglyceride; PL, phospholipid; CHO, cholesterol. The background color represents the correlation coefficient; one * represents P < 0.05 and two * represents P < 0.01. [file Image_6.JPEG]

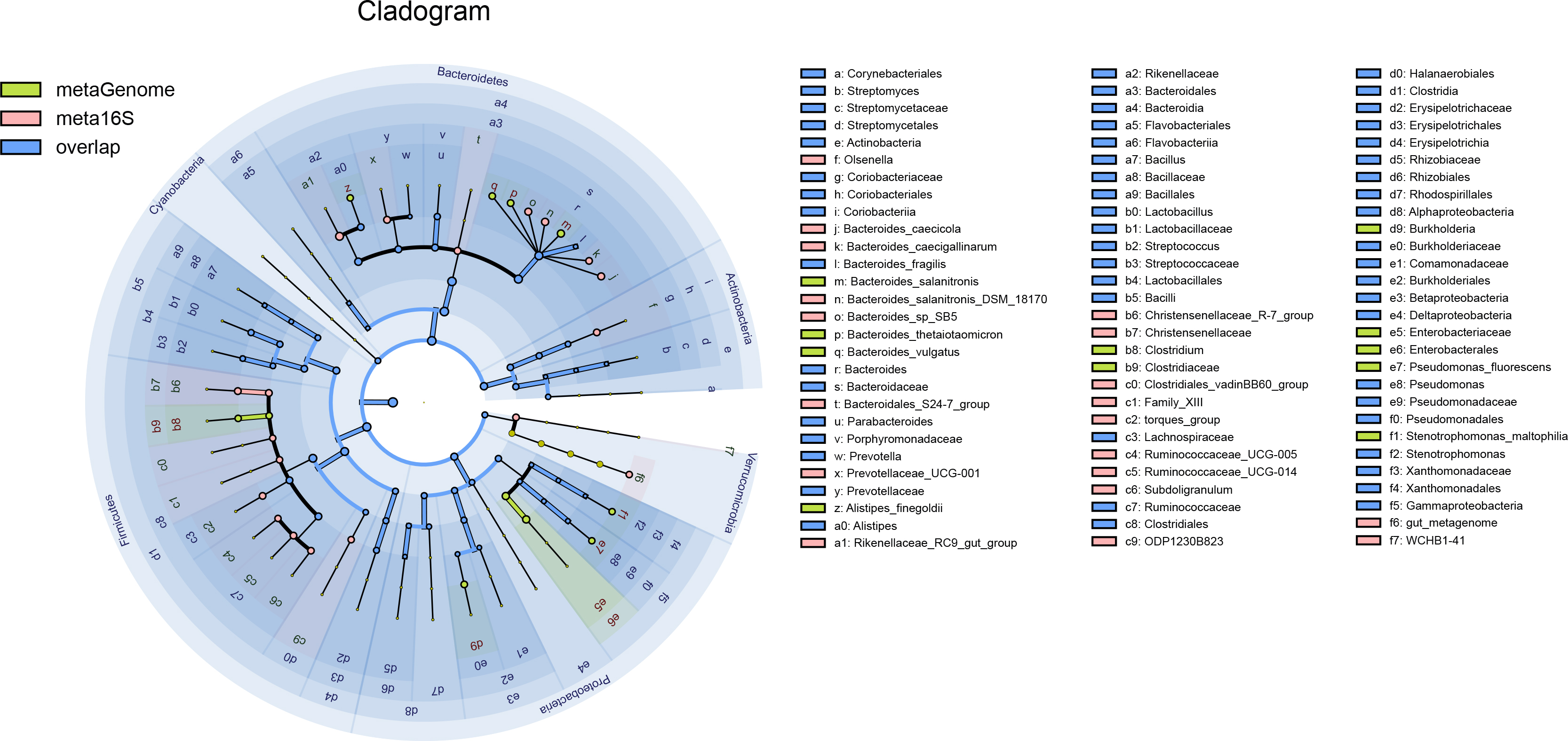

Supplement: Supplementary Figure 7 — The consistency of the results of the shotgun metagenomic and 16S rRNA gene sequencing. The small dots represent species, and the evolutionary branch tree represents the Kingdom, Phylum, Class, Order, Family, Genus and Species from the inside to the outside accordingly. The microorganisms with blue background were identified by both shotgun metagenomic sequencing and 16S rRNA gene sequencing, the microorganisms with green background were identified only by shotgun metagenomic sequencing, while the microorganisms with red background were identified only by 16S rRNA gene sequencing. The dot size indicates the average abundance of the microorganisms in all samples. [file Image_7.JPEG]

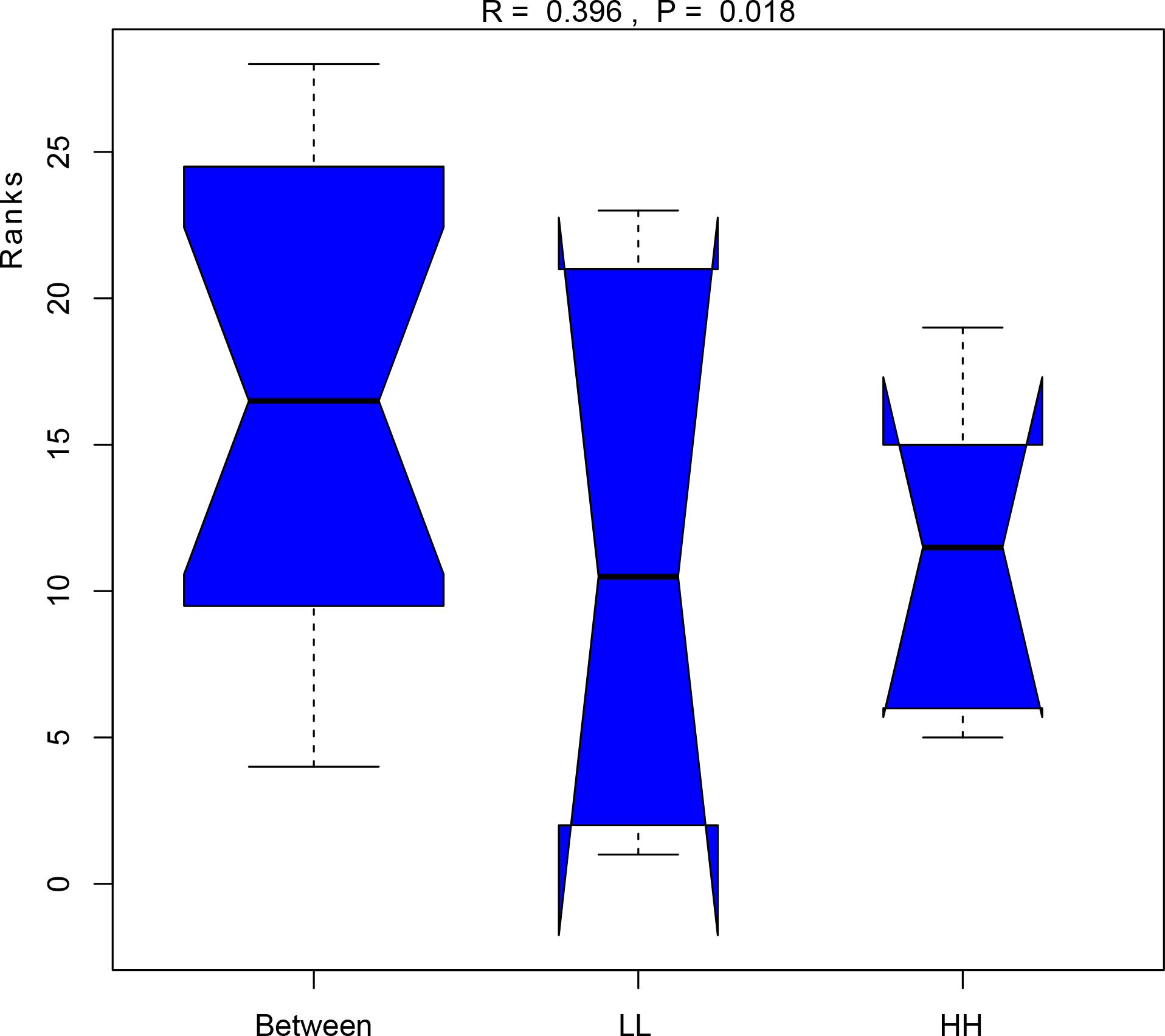

Supplement: Supplementary Figure 8 — The Anosim and Adonis comparison on Phylum composition between chickens with extreme abdominal fat deposition traits. HH, high AFP chickens; LL, low AFP chickens. [file Image_8.JPEG]

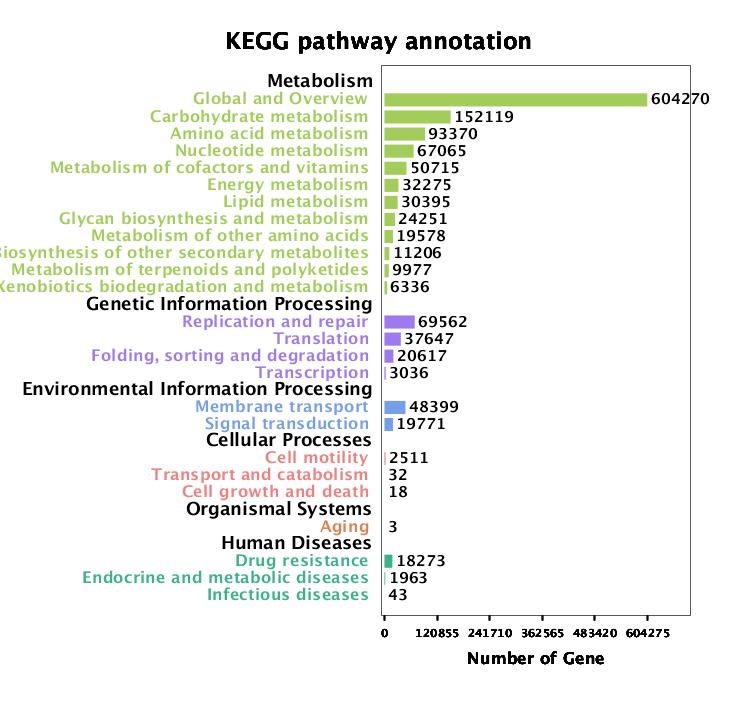

Supplement: Supplementary Figure 9 — Number of genes annotated to KEGG pathways for all samples. [file Image_9.PNG]

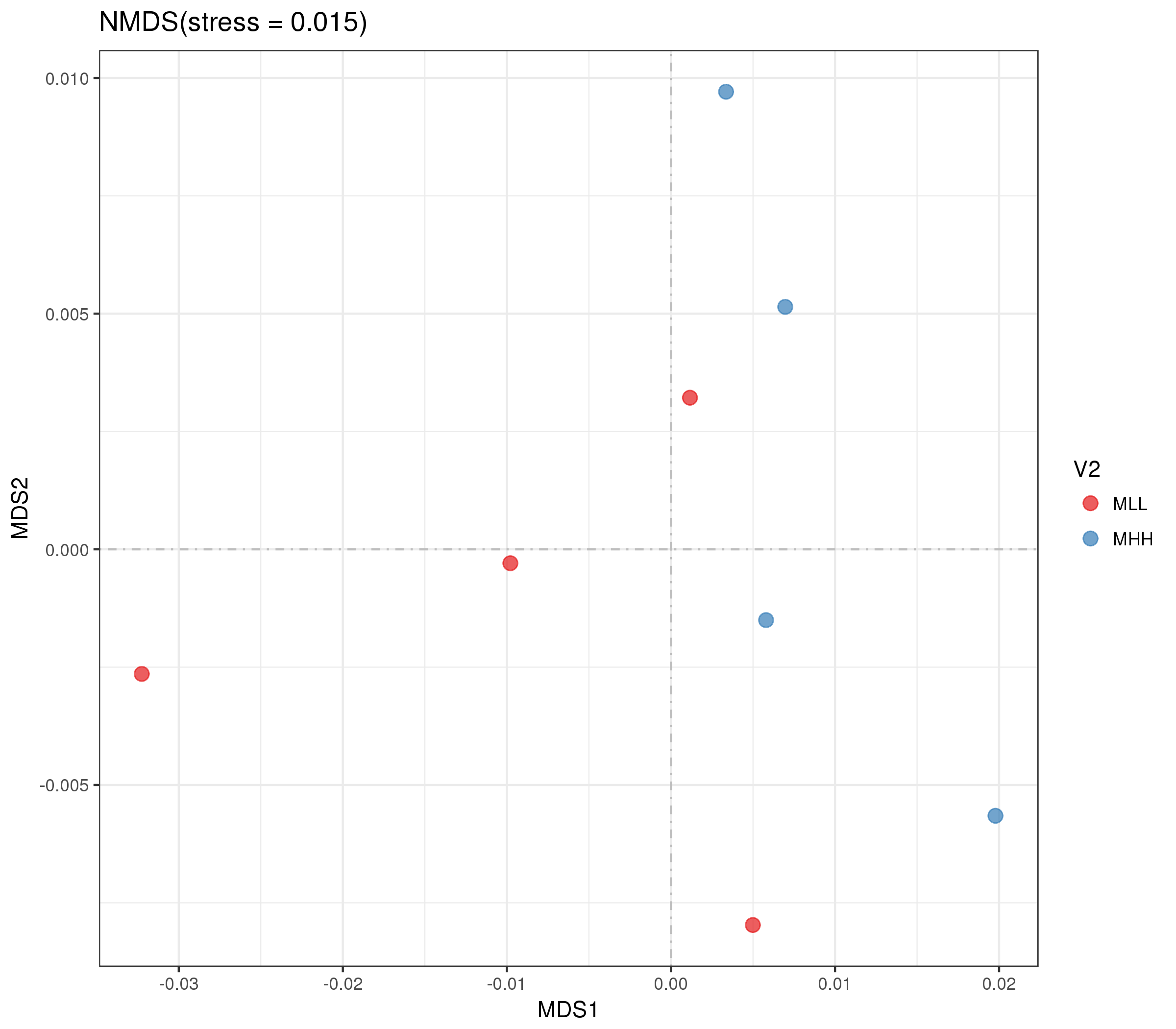

Supplement: Supplementary Figure 10 — NMDS analysis based on KEGG pathways. HH, high AFP chickens; LL, low AFP chickens. [file Image_10.PNG]

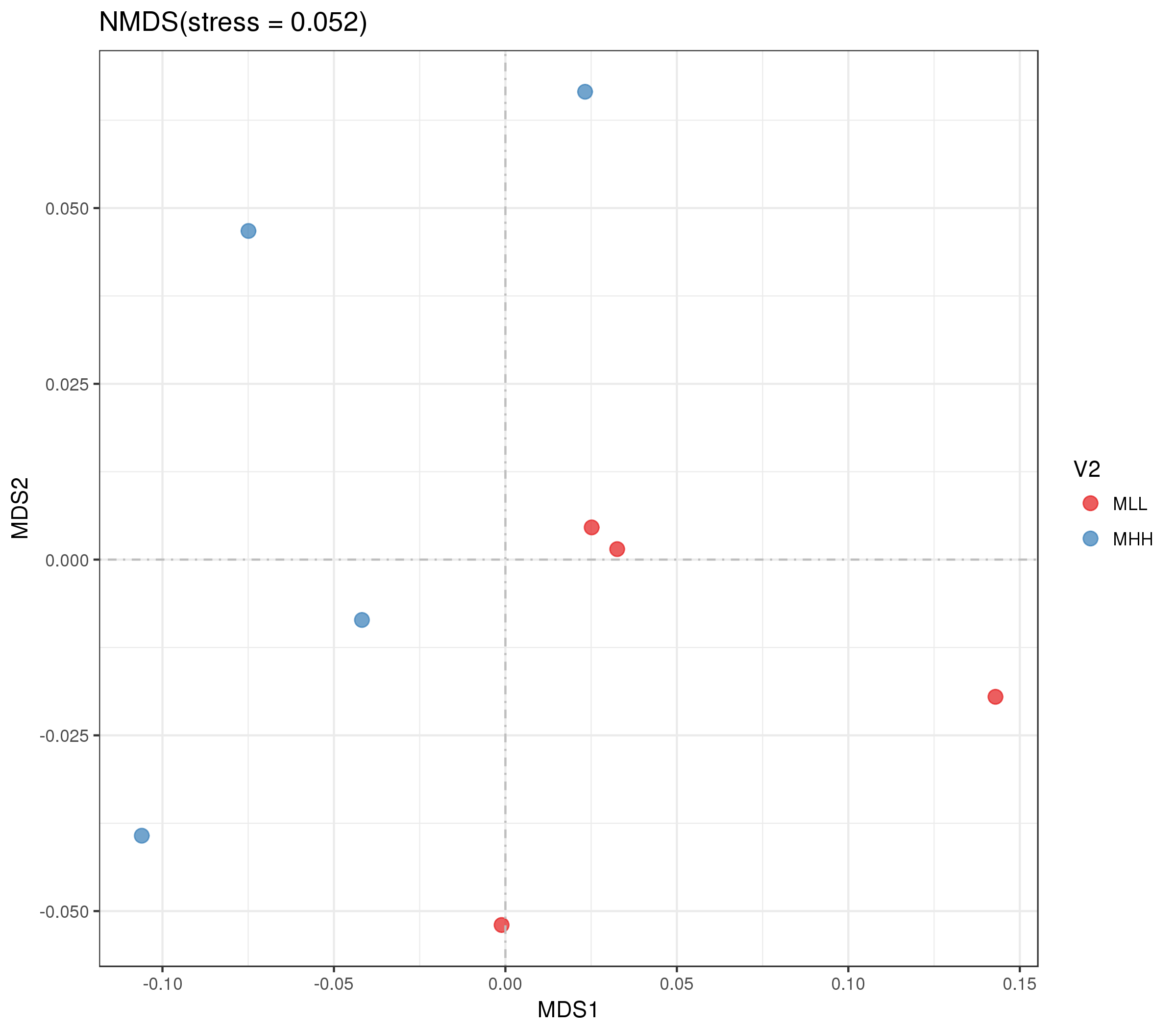

Supplement: Supplementary Figure 11 — NMDS analysis for eggNOG.C annotation. HH, high AFP chickens; LL, low AFP chickens. [file Image_11.PNG]

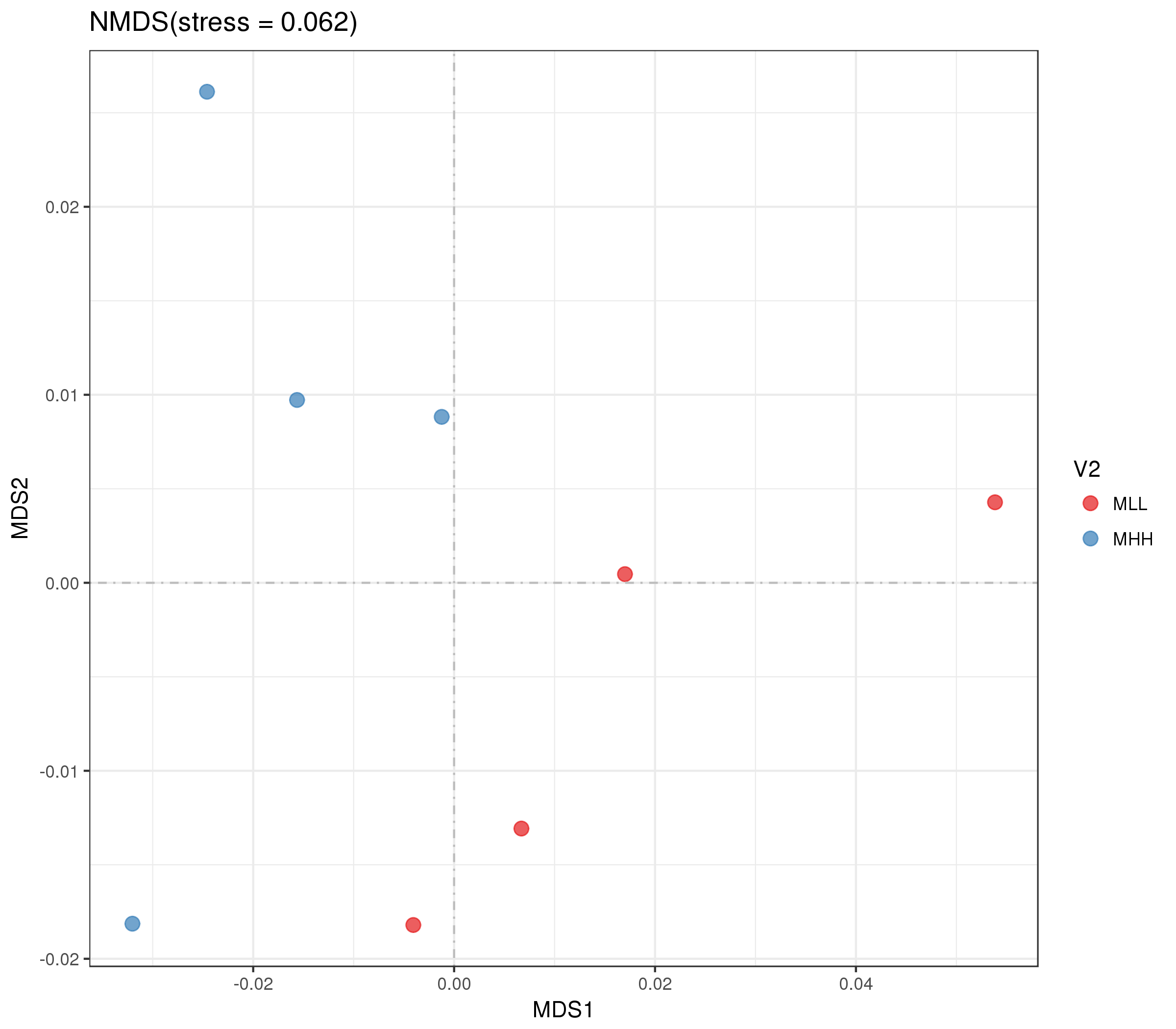

Supplement: Supplementary Figure 12 — NMDS analysis based on CAZy activities. HH, high AFP chickens; LL, low AFP chickens. [file Image_12.PNG]

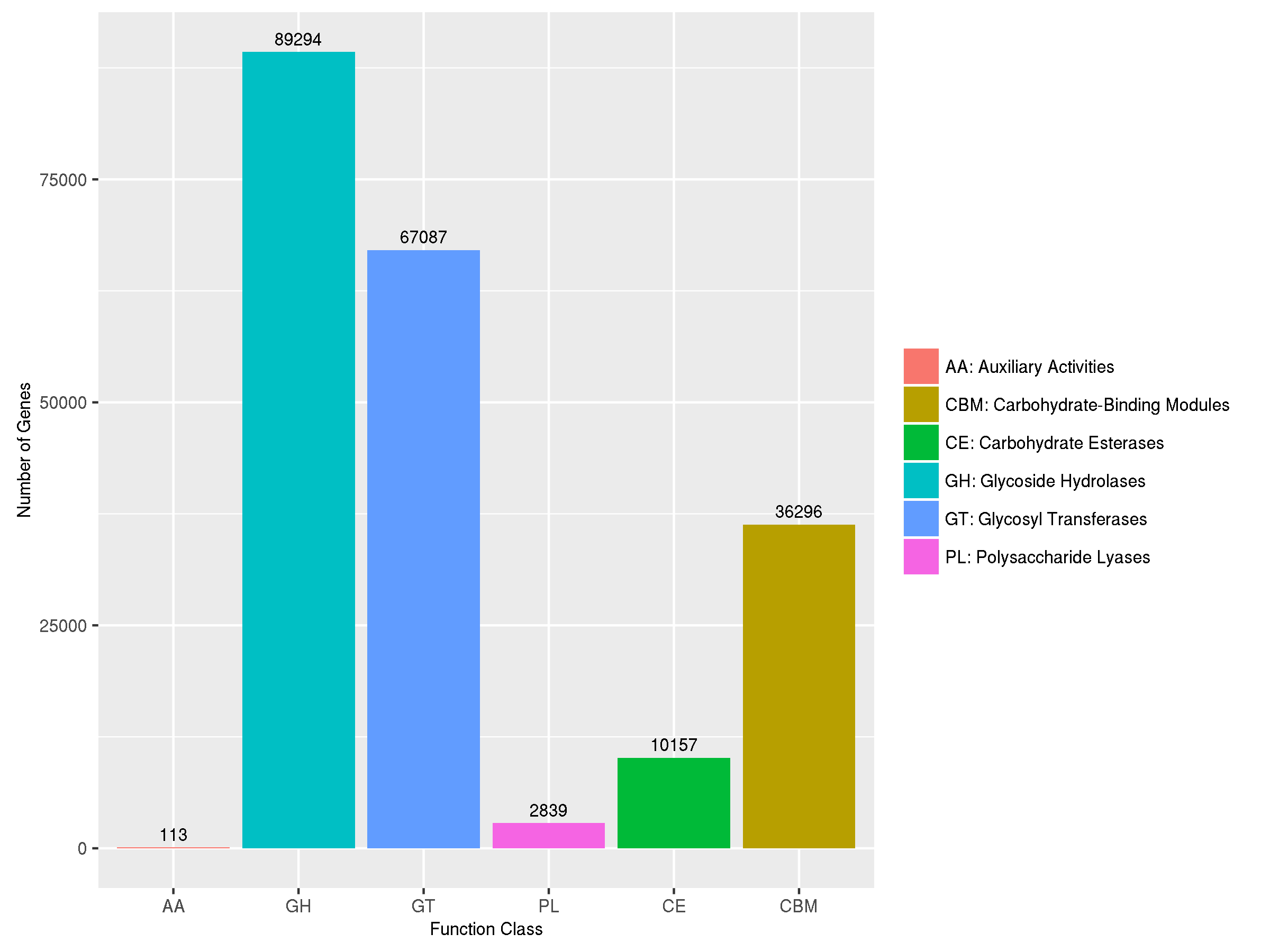

Supplement: Supplementary Figure 13 — Number of genes annotated to each CAZy activities for all samples. [file Image_13.PNG]
