# Supplementary material for: Specific Microbial Taxa and Functional Capacity Contribute to Chicken Abdominal Fat Deposition
Source: Front Microbiol. 2021 Mar 17;12:643025. doi: 10.3389/fmicb.2021.643025 (PMC8010200; doi:10.3389/fmicb.2021.643025)
Supplement: Supplementary file 14 [file Table_1.docx]

**Supplementary Table 1. The descriptive statistics of the abdominal fat deposition of Tiannong Partridge chickens**

| **Items** | **All chickens (n=400)** | **Top ten percent (n=40)** | **Last ten percent (n=40)** |
| --- | --- | --- | --- |
| BW (g) | 1513.31±181.78 | 1860.25±120.25 | 1239.25±55.26 |
| CW (g) | 1344.01±163.96 | 1661.60±118.64 | 1098.35±48.71 |
| EW (g) | 1024.36±127.11 | 1265.64±89.29 | 828.99±29.90 |
| AFW (g) | 43.54±16.91 | 77.52±11.18 | 19.15±4.14 |
| AFP (%) | 4.22±1.45 | 7.03±0.80 | 1.98±0.43 |
| IMF (%) | 1.97±0.76 | 3.57±0.46 | 0.91±0.28 |
| TG (mg/g) | 2.66±1.06 | 4.85±0.99 | 1.18±0.24 |
| CHO (mg/g) | 0.73±0.31 | 1.32±0.15 | 0.23±0.08 |
| PL (mg/g) | 0.22±0.08 | 0.42±0.09 | 0.14±0.01 |

HH, high AFP chickens; LL, low AFP chickens; BW, body weight; CW, carcass weight; EW, eviscerated weight; AFW, abdominal fat weight; AFP, abdominal fat percentage; IMF, intramuscular fat; TG, triglyceride; PL, phospholipid; CHO, cholesterol.

**Supplementary Table 2. Intergroup differences in abdominal fat deposition for 16S analyses.**

| **Group/Items** | **HH (n=38)** | **LL (n=38)** | ***P* value** | **Ratio (HH/LL)** |
| --- | --- | --- | --- | --- |
| BW (g) | 1582.63±187.06 | 1501.05±161.07 | 0.045 | 1.05 |
| CW (g) | 1375.21±186.13 | 1307.68±147.78 | 0.017 | 1.05 |
| EW (g) | 1036.63±147.81 | 990.79±119.18 | 0.016 | 1.05 |
| AFW (g) | 59.18±17.58 | 29.12±11.04 | 0.000 | 2.03 |
| AFP (%) | 5.67±1.28 | 2.89±1.00 | 0.000 | 1.97 |
| IMF (%) | 2.04±0.88 | 2.15±0.82 | 0.321 | 0.95 |
| TG (mg/g) | 3.05±1.13 | 2.52±0.85 | 0.210 | 1.21 |
| CHO (mg/g) | 0.83±0.30 | 0.71±0.37 | 0.638 | 1.17 |
| PL (mg/g) | 0.26±0.10 | 0.28±0.13 | 0.613 | 0.91 |

HH, high AFP chickens; LL, low AFP chickens; BW, body weight; CW, carcass weight; EW, eviscerated weight; AFW, abdominal fat weight; AFP, abdominal fat percentage; IMF, intramuscular fat; TG, triglyceride; PL, phospholipid; CHO, cholesterol.

**Supplementary Table 3. Intergroup differences in abdominal fat deposition for metagenome analyses.**

| **Group/Items** | **HH (n=4)** | **LL (n=4)** | ***P* value** | **Ratio (HH/LL)** |
| --- | --- | --- | --- | --- |
| BW (g) | 1775.00±266.52 | 1330.00±111.65 | 0.037 | 1.33 |
| CW (g) | 1548.75±249.48 | 1161.25±109.57 | 0.045 | 1.33 |
| EW (g) | 1191.28±196.62 | 906.60±79.39 | 0.167 | 1.17 |
| **AFW (g)** | **82.28±21.35** | **18.83±5.98** | **0.006** | **4.58** |
| **AFP (%)** | **7.23±1.24** | **2.08±0.63** | **0.001** | **3.47** |
| IMF (%) | 2.23±0.65 | 2.15±0.82 | 0.638 | 0.86 |
| TG (mg/g) | 2.37±0.62 | 2.52±0.85 | 0.649 | 1.15 |
| CHO (mg/g) | 0.60±0.11 | 0.71±0.37 | 0.788 | 1.04 |
| PL (mg/g) | 0.25±0.06 | 0.28±0.04 | 0.931 | 0.99 |

HH, high AFP chickens; LL, low AFP chickens; BW, body weight; CW, carcass weight; EW, eviscerated weight; AFW, abdominal fat weight; AFP, abdominal fat percentage; IMF, intramuscular fat; TG, triglyceride; PL, phospholipid; CHO, cholesterol.

**Supplementary Table 4. The differentially expressed** **orthologous groups between the high and low AFP chickens.**

| **Higher group** | **Labels** | **Function** | **Note** |
| --- | --- | --- | --- |
| LL | 4NE90 | Carbohydrate transport and metabolism | Belongs to the glycosyl hydrolase 3 family |
|  | COG0738 | Carbohydrate transport and metabolism | Major facilitator superfamily |
|  | COG4584 | Cell cycle control, cell division, chromosome partitionin | Plasmid stabilization system |
|  | COG0845 | Cell wall/membrane/envelope biogenesis | Belongs to the membrane fusion protein (MFP) (TC 8.A.1) family |
|  | COG1143 | Energy production and conversion | NDH-1 shuttles electrons from NADH, via FMN and iron- sulfur (Fe-S) centers, to quinones in the respiratory chain. The immediate electron acceptor for the enzyme in this species is believed to be ubiquinone. Couples the redox reaction to proton translocation (for every two electrons transferred, four hydrogen ions are translocated across the cytoplasmic membrane), and thus conserves the redox energy in a proton gradient |
|  | 2FM14 | Function unknown | Psort location Cytoplasmic, score 8.96 |
|  | COG5464 | Function unknown | double-stranded DNA endodeoxyribonuclease activity |
|  | 4P1Z5 | Inorganic ion transport and metabolism | TonB-linked outer membrane protein, SusC RagA family |
|  | 4NDXS | Inorganic ion transport and metabolism | TonB-linked outer membrane protein, SusC RagA family |
|  | COG0823 | Intracellular trafficking, secretion, and vesicular transport | Involved in the tonB-independent uptake of proteins |
|  | COG0776 | Replication, recombination and repair | regulation of translation |
|  | COG1193 | Replication, recombination and repair | negative regulation of DNA recombination |
|  | 4NEY6 | Replication, recombination and repair | Integrase core domain |
|  | 2FMHY | Replication, recombination and repair | COG4584 Transposase and inactivated derivatives |
|  | COG0514 | Replication, recombination and repair | ATP-dependent DNA helicase (RecQ) |
| HH | COG0768 | Cell wall/membrane/envelope biogenesis | penicillin binding |
|  | COG1475 | Transcription | chromosome segregation |
|  | COG1609 | Transcription | purine nucleotide biosynthetic process |

HH, high AFP chickens; LL, low AFP chickens.

**Supplementary Table 5. The differentially expressed** **CAZy activities between the high and low AFP chickens.**

| **Family** | **Labels** | **LL** | **HH** | **Main Functionalities/Kinetics and Mechanism** |
| --- | --- | --- | --- | --- |
| Carbohydrate-Binding Module | CBM13 | 1030.88 | 1111.45 |  |
|  | CBM34 | 103.58 | 153.48 |  |
|  | CBM37 | 63.82 | 106.43 |  |
|  | CBM50 | 6097.26 | 6491.44 | CBM50 modules are generally found in bacterial lysins including muramidase, N-acetylglucosaminidase, γ-D-glutamate-meso-diaminopimelate muropeptidase and N-acetylmuramoyl-L-alanine amidase. The CBM50 modules in lysins are shown to bind to bacterial peptidoglycan and involved in cell division by localizing these enzymes to the divisional site. CBM50 modules were also found in family GH18 chitinases and contribute to the antifungal activity of the enzymes through their binding ability to chitinous component of the fungal cell wall. |
| Glycoside Hydrolase | GH42 | 142.08 | 175.48 | GH42 enzymes are active on lactose and transgalactosylation was observed with production of galactooligosaccharides. However, several GH42 enzymes are extracted from diverse habitats where lactose would not be present and they are very active on galactooligosaccharides and galactans, suggesting that these enzymes would be involved in vivo in plant cell wall degradation. The activity of GH42 enzymes on lactose and lactulose has interesting potential for the removal of the former from dairy products and to monitor lactulose concentration during heat treatment leading to UHT milk. Family GH42 β-galactosidase are retaining enzymes and follow the classical Koshland double-displacement mechanism. |
|  | GH49 | 24.32 | 40.23 | Glycoside hydrolases of family 49 cleave α-1,6-glucosidic linkages or α-1,4-glucosidic linkages of polysaccharides containing α-1,6-glucosidic linkages, dextran, and pullulan. The major activities reported for this family of glycoside hydrolases are dextranase (EC 3.2.1.11), and a dextranase from Penicillium minioluteum, Dex49A, is currently the most characterised enzyme. Family GH49 α-glycosidases are inverting enzyme. |
| Glycosyl Transferase | GT39 | 46.31 | 66.02 | These enzymes use dolichol-P-mannose (b-linked) as the sugar donor. |
|  | GT66 | 272.25 | 307.39 | These enzymes utilize a lipid-diphospho-oligosaccharide as the donor. |
|  | GT7 | 170.91 | 203.91 |  |
| Carbohydrate Esterase | CE2 | 93.30 | 68.12 |  |
|  | CE6 | 262.40 | 199.11 |  |
| Glycoside Hydrolase | GH10 | 366.58 | 290.53 | Family GH10 xylanases are retaining enzymes and follow a classical Koshland double-displacement mechanism. |
|  | GH109 | 460.73 | 399.27 | Family GH109 enzymes operate via the unusual NAD-dependent hydrolysis mechanism involving an NAD+ cofactor, that so far has been seen only in Glycoside Hydrolase Family 4 (GH4), despite different overall folds between these families. NMR monitoring of the reaction catalyzed by α-N-acetylgalactosaminidase indicated that the enzyme proceeds with retention of the anomeric configuration and concomitant exchange of the GalNAc H-2 atom for a solvent proton. This, and the indispensable presence of NAD+, indicate that GH109 enzymes most likely operate by a similar retaining mechanism. The mechanism of GH109 enzymes allows cleavage of thioglycosides and of glycosides of the opposite anomeric configuration (both at a comparatively slow rate), two features that are extremely rare among 'classical' glycosidases. |
|  | GH11 | 61.37 | 48.42 | The glycoside hydrolases of this family are endo-β-1,4-xylanases. No other activities have been observed. As a historical note, GH11 was one of the first glycoside hydrolase families classified by sequence analysis and was previously known as "Cellulase Family G" prior to extensive enzymological characterization. Family GH11 xylanases are retaining enzymes and follow a classical Koshland double-displacement mechanism. |
|  | GH146 | 106.66 | 71.93 | Enzymes of this family of glycoside hydrolases exhibit β-arabinofuranosidase activity. The founding member of this family, BT0349 from Bacteroides thetaiotaomicron, cleaves both β1,2- and β1,3-linked arabinofuranose side chains present in branched sugar beet arabinan. BT0349 β-arabinofuranosidase displays exo-activity on β-linked arabinofuranosyl groups and has been proposed to act with a retaining mechanism, based on the positions of the catalytic residues. |
|  | GH16 | 1993.26 | 1693.19 | The members of family 16 are active on β-1,4 or β-1,3 glycosidic bonds in various glucans and galactans. A wide diversity of glycoside hydrolases active on plant and marine polysaccharides are found in GH16. Members of GH16 enzymes are retaining enzymes, as first shown by NMR on an endo-1,3-1,4-β-D-glucan 4-glucanohydrolase from Bacillus licheniformis. As such, they utilize a covalent glycosyl-enzyme intermediate, which is broken-down by glycosyl transfer to water or a carbohydrate acceptor substrate in glycoside hydrolases or transglycosylases, respectively. |
|  | GH29 | 1107.47 | 914.77 | The glycoside hydrolases of this family are exo-acting α-fucosidases from archaeal, bacterial, and eukaryotic origin. No other activities have been observed for GH29 family members. GH29 α-fucosidases are retaining enzymes following a classical Koshland double-displacement mechanism. |
|  | GH30 | 197.31 | 162.59 | This family contains glycoside hydrolases with three known enzyme activities: β-glucosylceramidase, β-1,6-glucanase, and β-xylosidase. This family currently contains enzymes from only bacteria and eukaryotes. Family GH30 enzymes are retaining enzymes and follow the classic Koshland double-displacement mechanism. The β-glucosylceramidases require an activator protein and negatively charged phospholipids for optimal activity. Neither the β-1,6-glucanases nor the β-xylosidases appear to require any activators. |
|  | GH35 | 367.64 | 301.73 | Most glycoside hydrolases of GH35 are β-galactosidases (EC 3.2.1.23). GH35 enzymes have been isolated from microorganisms such as fungi, bacteria, and yeasts, as well as higher organisms such as plants, animals, and human cells. These β-galactosidases catalyse the hydrolysis of terminal non-reducing β-D-galactose residues in, for example, lactose (1,4-O-β-D-galactopyranosyl-D-glucose), oligosaccharides, glycolipids, and glycoproteins. Various GH35 β-galactosidases demonstrate specificity towards β-1,3-, β-1,6- or β-1,4-galactosidic linkages, and are often most active under acidic conditions. |
|  | GH67 | 57.28 | 35.61 | Glycoside hydrolases of this family display alpha-glucuronidase activity. The enzymes target the glucuronic acid appended to the C2-OH of the xylose at the non-reducing end of xylooligosaccharides. The enzymes display a preference for 4-O-methyl-D-glucuronic acid side chains. These enzymes do not remove glucuronic acid from internal regions of xylan. The enzymes are generally intracellular, or membrane associated suggesting that they play a terminal role in uncapping decorated xyloooligosaccharides, making these molecules available to beta-xylosidases produced by the host. α-glucuronidases are inverting enzymes that hydrolyse their target glycoside bond through a single displacement mechanism assisted by general acid and general base residues. Thus, the glucuronic acid is formed in a beta configuration. |
| Glycosyl Transferase | GT11 | 80.43 | 50.80 |  |
|  | GT3 | 223.29 | 198.04 |  |
|  | GT30 | 493.00 | 421.33 | These enzymes use a nucleotide monophosphosugar as the donor (CMP-b-KDO) instead of a nucleotide diphosphosugar |
| Polysaccharide Lyase | PL0 | 101.22 | 73.93 |  |
|  | PL33 | 64.67 | 41.49 |  |

HH, high AFP chickens; LL, low AFP chickens.
